# Time‐integrated δ^2^ H in *n*‐alkanes and carbohydrates from boreal needles reveal intra‐annual physiological and environmental signals

**DOI:** 10.1111/nph.20448

**Published:** 2025-02-21

**Authors:** Charlotte Angove, Guido L. B. Wiesenberg, Marco M. Lehmann, Matthias Saurer, Yu Tang, Elina Sahlstedt, Tatjana C. Speckert, Pauliina P. Schiestl‐Aalto, Katja T. Rinne‐Garmston

**Affiliations:** ^1^ Stable Isotope Laboratory of Luke (SILL) Natural Resources Institute Finland (Luke) Helsinki 00790 Finland; ^2^ University of Helsinki Helsinki 00790 Finland; ^3^ Department of Geography University of Zürich Zürich 8057 Switzerland; ^4^ Forest Soils and Biogeochemistry, Swiss Federal Institute for Forest Snow and Landscape Research (WSL) Birmensdorf 8903 Switzerland; ^5^ College of Urban and Environmental Sciences Peking University Beijing 100871 China; ^6^ Faculty of Science, Institute for Atmospheric and Earth System Research (INAR)/Physics University of Helsinki Helsinki 00014 Finland

**Keywords:** bioindicators, deuterium, forest, isotopes, leaf sugar, leaf water evaporative enrichment, lipids, paleohydrology

## Abstract

- Reliable insights from key δ^2^H bioindicators, *n*‐alkanes and carbohydrates, are hindered by our limited understanding of isotope fractionation processes related to leaf water and primary assimilates. We addressed this with the first study to investigate time‐integrated intra‐annual δ^2^H signals of *n*‐alkanes and carbohydrates in a natural forest.
- We sampled 1‐yr‐old needles (1N) and current‐year needles (0N) from five Scots Pine trees in a coniferous forest in Hyytiälä, Finland, biweekly during 2019. The δ^2^H of their *n*‐alkanes (δ^2^H_alkane_), water‐soluble carbohydrates (δ^2^H_WSC_) and starch (δ^2^H_starch_) were evaluated for time‐integrated physiological (gas‐exchange) and environmental (hydrological) signals.
- Time integration was critical for interpreting δ^2^H_WSC_ and δ^2^H_alkane_. Time‐integrated net assimilation rate (*T*:*A*_n_), the strongest signal in δ^2^H_WSC_, correlated negatively with 1N δ^2^H_WSC_ (early season) and positively with 0N δ^2^H_WSC_ (late season). Unexpectedly, δ^2^H_alkane_ exhibited stronger environmental signals in 1N than in 0N, with the most pronounced being physiologically mediated hydrological signals.
- *T*:*A*_n_ is a major signal in intra‐annual δ^2^H_WSC_ in a boreal forest, subject to seasonal interactions with needle age and δ^2^H_starch_. There could be enough *de novo n*‐alkane synthesis *in situ* to enhance the reliability of δ^2^H_alkane_ as a hydrological indicator, bringing promise to interpretations of the *n*‐alkane palaeohydrological record.

## Introduction

The stable H isotope ratio (δ^2^H) in plant‐derived biomarkers (e.g. *n*‐alkanes, tree‐ring cellulose) has proven value, and profound potential, for insights into paleoenvironmental reconstructions and plant stress response (Dawson *et al*., 2004; Kahmen *et al*., 2013; Lehmann *et al*., 2024a). A key barrier that limits their reliability and implementation is that their H isotope composition can be affected by multiple isotope fractionating (Zhu *et al*., 2020; Holloway‐Phillips *et al*., 2022; Baan *et al*., 2023a) and nonfractionating (i.e., mixing; Liu *et al*., 2021) processes. This can interfere with meaningful environmental or physiological insights from δ^2^H values in plant bioindicators (Baan *et al*., 2023b). Undoubtedly, they will benefit from a thorough examination of how the δ^2^H of plant compounds correlates with environmental and physiological signals under natural conditions.

### Background

*n*‐Alkanes with chain lengths of 25–35 carbons are straight‐chained hydrocarbons found in leaf epicuticular waxes, which can be key contributors to the δ^2^H of *n*‐alkanes (δ^2^H_alkane_, Table 1) in soils, used to interpret past climate (Dawson *et al*., 2004; Schefuß *et al*., 2005; Thomas *et al*., 2021). Overall, δ^2^H_alkane_ acts as an indicator of δ^2^H in precipitation (δ^2^H_precip_), modified by leaf evaporative enrichment, local meteorological factors (i.e. evapotranspiration (ET) and relative humidity (RH)) and source water (Sachse *et al*., 2006), which is further modified by biochemical isotope fractionation (Newberry *et al*., 2015; Baan *et al*., 2023a,b). δ^2^H_alkane_ in leaves can be related to δ^2^H in leaf water (δ^2^H_l‐water_; Freimuth *et al*., 2017; Zhu *et al*., 2020; Lehmann *et al*., 2024b), though not necessarily (McInerney *et al*., 2011), and evidence from seasonal δ^2^H_alkane_ variability of new needles in a natural forest shows that the δ^2^H_l‐water_ signal can be obscured, potentially by changes in carbohydrate sourcing throughout the season (Newberry *et al*., 2015). Furthermore, the δ^2^H_alkane_ in new leaf tissue can record a shift from heterotrophy to autotrophy (Tipple & Ehleringer, 2018), and if leaves are sampled before autotrophy has been fully established, then the δ^2^H_l‐water_ signal may be obscured (Zhu *et al*., 2020).

**Table 1:** Abbreviations and symbols used in the text.

Abbreviation	Description
NADPH	Nicotinamide adenine dinucleotide phosphate
NSC	Nonstructural carbohydrates
WSC	Water‐soluble carbohydrates
δ^2^H	Isotope ratio of ^2^H compared to ^1^H, relative to VSMOW (‰)
δ^2^H_alkane_	δ^2^H of *n*‐alkanes
δ^2^H_precip_	δ^2^H of precipitation
δ^2^H_l‐water_	δ^2^H of leaf water
δ^2^H_n‐water_	δ^2^H of needle water
δ^2^H_starch_	δ^2^H of starch
δ^2^H_WSC_	δ^2^H of water‐soluble carbohydrates
δ^2^H_source_	δ^2^H of source water
δ^2^H_vapor_	δ^2^H of water vapor
δ^18^O	Isotope ratio of ^18^O compared to ^16^O, relative to VSMOW (‰)
Δ^2^H_n‐water_	Needle water ^2^H enrichment above source (twig) water (‰)
ɛ_bio_	Hydrogen isotope offset between *n*‐alkanes and leaf water
RH	Atmospheric relative humidity
*E*	Transpiration rate
*A*_n_	Net assimilation rate
*g*_s_	Stomatal conductance
*T*:RH	Time‐integrated atmospheric relative humidity
*T*:*E*	Time‐integrated transpiration rate
*T*:*A*_n_	Time‐integrated net assimilation rate
*T*:*g*_s_	Time‐integrated stomatal conductance
*T*:δ^2^H_n‐water_	Time‐integrated δ^2^H of needle water
*T*:∆^2^H_n‐water_	Time‐integrated needle water ^2^H enrichment above source (twig) water
*T*:δ^2^H_source_	Time‐integrated δ^2^H of source water
0N	Current‐year needles
1N	One‐year‐old needles
*R* ^2^(M)	Marginal *R* ^2^. A pseudo‐*R* ^2^ estimate for the models being tested
*R* ^2^(C)	Conditional *R* ^2^. A pseudo‐*R* ^2^ estimate for the models being tested combined with model random effects, such as sampling date, time and site.
ICC	Intraclass correlation. The probability that two values from the same sampling date, and/or tree identity, correlate, on a scale of 0–1.

Interspecies differences in leaf δ^2^H_alkane_ are more constant between 2 yr than for leaf cellulose δ^2^H, suggesting that species differences in biosynthetic fractionation for δ^2^H_alkane_ are likely the more invariable and less sensitive to environmental variability (Baan *et al*., 2023a). Meanwhile, at the scale of intraleaf variability, δ^2^H_alkane_ is likely more environmentally sensitive than leaf cellulose δ^2^H, owing to a higher proportion of autotrophic production during *n*‐alkane syntheses (Zhu *et al*., 2020). Leaf nonstructural carbohydrates, such as sugars (e.g. sucrose and glucose) and starch, are key intermediaries in the transfer of the δ^2^H signal from leaf water to cellulose (Holloway‐Phillips *et al*., 2022; Lehmann *et al*., 2022), as observed for carbon‐13 (δ^13^C) and oxygen‐18 (δ^18^O) (Gessler *et al*., 2009). It is valuable to understand their role as δ^2^H intermediaries between leaf water and tree rings because tree‐ring δ^2^H can correlate with δ^2^H_l‐water_ (Lehmann *et al*., 2024a). Yet, prevailing intra‐annual physiological or climatic signals from δ^2^H_WSC_ and δ^2^H_starch_ has not yet been explored in a natural forest. Evidence from a ^2^H‐enrichment study shows that the δ^2^H in leaf sucrose is likely determined by physiological processes (Augusti *et al*., 2006). Further evidence shows the ^2^H offset between leaf sucrose and water having a variable relationship with δ^2^H_l‐water_, in addition to weak relationships with dark respiration, sugar pool turnover time and the proportion of sugar in the sugar and starch pool (Holloway‐Phillips *et al*., 2022). These trends were reinforced by results from the δ^2^H of water‐soluble carbohydrates (WSCs), a mixture of sugars and sugar alcohols, suggesting that δ^2^H in leaf sugars is determined by the relative concentrations of sugars and starch, and leaf gas exchange (Lehmann *et al*., 2024b). It is thus valuable to observe whether these signals exhibited in glasshouse experiments (Holloway‐Phillips *et al*., 2022; Lehmann *et al*., 2024b) can also be observed in intra‐annual variation of a natural forest. Water‐soluble carbohydrates are used as a proxy for leaf sugars (Leppä *et al*., 2022; Tang *et al*., 2022; Lehmann *et al*., 2024b), as the simplest bulk matter extract that can be measured without using compound‐specific isotope analysis. We hereafter use leaf WSC δ^2^H (δ^2^H_WSC_) as a proxy for leaf sugar δ^2^H. This comes at the disadvantage of measuring a diverse group of leaf compounds with different metabolic history.

Even though leaf carbon partitioning between sucrose and starch is likely instrumental to δ^2^H in sugars and tree‐ring cellulose (Holloway‐Phillips *et al*., 2022; Wieloch *et al*., 2022; Lehmann *et al*., 2024a), the δ^2^H of starch (δ^2^H_starch_) has not been compared with physiological or environmental trends. δ^2^H_starch_ is distinctly different from δ^2^H of sugars (Schleucher *et al*., 1999); therefore, it is important to investigate whether δ^2^H_starch_ has different physiological or climatic trends compared with δ^2^H in sugars because the temporally variable interaction between the starch and sugar pool may introduce mixed physiological or environmental signals absent from freshly assimilated sugars.

Hydrogen atoms in leaf carbohydrates (e.g. sugars and starch) and *n*‐alkanes are derived from the same leaf's water, but their δ^2^H are different, owing to their distinct biochemical pathways (Schleucher *et al*., 1999). For example, among the prominent hydrogen isotope‐fractionating processes that can lead to their different δ^2^H (Lehmann *et al*., 2024b), they can source different amounts of H atoms from nicotinamide adenine dinucleotide phosphate (NADPH), and these H atoms from NADPH can be a product of different NADPH‐synthesis pathways (Sessions *et al*., 1999; Zhou *et al*., 2016; Wijker *et al*., 2019). Furthermore, their precursor molecule sources can vary between photosynthetic and glycolytic origins and differently represent short‐term and long‐term storage (Cormier *et al*., 2018; Zhu *et al*., 2020; Lehmann *et al*., 2024b). Further, nonfractionating (i.e., mixing) processes likely further affect carbohydrate and *n*‐alkane δ^2^H differently, owing to their different metabolic fates – and these are particularly relevant in field studies (e.g. Leppä *et al*., 2022). The combined consequences of isotope‐fractionating and nonfractionating processes, to δ^2^H_alkane_ and leaf carbohydrate δ^2^H, are poorly understood. Therefore, it is highly valuable to investigate δ^2^H_alkane_ and leaf carbohydrate δ^2^H together, to elucidate their physiological and environmental information, enhancing our understanding of the relative importance of their different drivers.

### Temporal integration perspectives

A key step towards elucidating intra‐annual physiological and environmental signals from leaf δ^2^H_alkane_, δ^2^H_WSC_ and δ^2^H_starch_ *in situ* is quantifying important temporal aspects that could otherwise interfere with finding prevailing trends. Time integration has been long‐established in the interpretation of δ^18^O in organic compounds, especially tree rings (Gessler *et al*., 2009; Pérez‐de‐Lis *et al*., 2022), and it is known to be important for the transfer of the δ^18^O signal from leaf water to foliar water‐soluble organic matter (Barnard *et al*., 2007), WSC (Leppä *et al*., 2022) and resin (Tang *et al*., 2024). Therefore, it is long overdue to determine whether time‐integrated, intraseasonal signals are revealed from δ^2^H_alkane_, δ^2^H_WSC_ and δ^2^H_starch_. This is outstandingly relevant for δ^2^H_alkane_ because *n*‐alkanes are gradually accumulated during leaf development (Jetter *et al*., 2000). Indeed, leaf wax measurements from the evergreen shrub *Prunus laurocerasus* have shown that the leaf wax layer continues to thicken after leaf expansion; there were 10 molecular layers in 10‐d‐old leaves during expansion, *c*. 20 molecular layers after 50 d, and 1‐yr‐old leaves had 35–45 layers (Jetter *et al*., 2000; Jetter & Schäffer, 2001). Given that new leaf tissue growth coincides with the predominant wax production, it is a reasonable assumption that the leaf δ^2^H_alkane_ largely represents only the early stages of the leaf lifespan (Kahmen *et al*., 2011; Sachse *et al*., 2015; Freimuth *et al*., 2017). Other recent studies argue for the continuous formation of *n*‐alkanes in leaves during the growing season (Speckert *et al*., 2023) and rapid response of *n*‐alkane formation as a response to environmental stress such as drought, without increased concentration of *n*‐alkanes in the wax layer (Srivastava & Wiesenberg, 2018). Since temperature and elevated CO_2_ can affect the composition of *n*‐alkanes and their precursors, fatty acids, within leaves (Ofiti *et al*., 2023), the hydrological signal in δ^2^H_alkane_ may not only be obscured by integration time but also the variability in conditions exposed to leaves during their integration.

Evidence from WSC δ^18^O would suggest that the δ^2^H_WSC_ integration period during a growing season can vary from within 48 h to more than 5 d (Leppä *et al*., 2022). Meanwhile, the δ^2^H_starch_ integration period could depend on the relative contribution of transitory starch to the total leaf starch pool. If the leaf starch pool is mostly transitory, the δ^2^H_starch_ integration period will likely be 1 d (Weise *et al*., 2011; Fernandez *et al*., 2017).

### Spatiotemporal integration perspectives

δ^2^H_l‐water_ becomes higher with increased proximity to evaporative sites (Luo & Sternberg, 1992; Gan *et al*., 2002; Farquhar & Gan, 2003). This phenomenon has demonstrated relevance for leaf spatial patterns in δ^2^H of cellulose and δ^2^H_alkane_ (Zhu *et al*., 2020; Liu *et al*., 2021). Since photosynthetic tissue water can be more exposed to evaporative ^2^H enrichment than bulk δ^2^H_l‐water_, leaf water isotope heterogeneity could be relevant for leaf δ^2^H_WSC_, δ^2^H_starch_ and δ^2^H_alkane_, like it has been reported for δ^18^O in sucrose (Baca Cabrera *et al*., 2023). Its role could be profound because the δ^2^H of source water (δ^2^H_source_) and evaporative ^2^H enrichment that make up δ^2^H_l‐water_ are distinctly different (Cernusak *et al*., 2016); therefore, an increased ratio of evaporative ^2^H enrichment could substantially change the δ^2^H of surrounding water during *n*‐alkane and carbohydrate syntheses. Resultantly, it is not clear whether the long‐term δ^2^H_WSC_, δ^2^H_starch_ and δ^2^H_alkane_ signals are more reflective of needle water δ^2^H (δ^2^H_n‐water_) or the ^2^H enrichment of needle water above source water (∆^2^H_n‐water_). Investigating the relative contributions of δ^2^H_n‐water_ and ∆^2^H_n‐water_ to δ^2^H_WSC_, δ^2^H_starch_ and δ^2^H_alkane_ in natural environments is crucial because it determines the ecohydrological conditions that they represent during palaeohydrological reconstructions.

### Study aim and hypotheses

We investigated whether there are time‐integrated, physiological or environmental signals in the variability of δ^2^H_alkane_, δ^2^H_WSC_ and δ^2^H_starch_ using a comprehensive seasonal field survey of *Pinus sylvestris* L. (Scots Pine). We hypothesized that (1) δ^2^H_alkane_ and δ^2^H_WSC_ have stronger relationships to physiological (leaf gas exchange) or environmental factors (e.g. RH and modeled water isotopes) after accounting for multiple integration days or weeks, and (2) δ^2^H_alkane_, δ^2^H_WSC_ and δ^2^H_starch_ are more strongly related to time‐integrated ∆^2^H_n‐water_ than δ^2^H_n‐water_ because they can be sensitive to inhomogeneities in leaf water (Zhu *et al*., 2020; Liu *et al*., 2021), and photosynthetic tissue water is likely more exposed to evaporative enrichment than bulk leaf water (Baca Cabrera *et al*., 2023). Finally, we hypothesized that (3) based on evidence that δ^2^H_alkane_ is related to δ^2^H_l‐water_ in controlled conditions (Freimuth *et al*., 2017; Lehmann *et al*., 2024b), enhanced by high autotrophic production in the field (Zhu *et al*., 2020), δ^2^H_alkane_ will reflect δ^2^H_l‐water_ while δ^2^H_WSC_ and δ^2^H_starch_ will represent leaf gas exchange (Holloway‐Phillips *et al*., 2022; Lehmann *et al*., 2024b).

## Materials and Methods

### Field site and sampling procedure

The study site, Hyytiälä Forest (61°51′N, 24°17′E), is a managed forest in central Finland, within the southern boreal vegetation zone. It is dominated by Scots pine (*Pinus sylvestris* L.), 56 yr old during sampling, and other species included Norway spruce (*Picea abies* (L.) H. Karst), birch (*Betula pendula* Roth, *Betula pubescens* Ehrh) and European aspen (*Populus tremula* L.) (Kolari *et al*., 2022). In 2018, there were 1304 trees ha^−1^, and the mean tree height was 19.9 m, with dominant tree height reaching 23.5 m (Kolari *et al*., 2022). The soil, which is a Haplic Podzol, is predominantly < 1 m deep, but there are moist depressions with a thicker layer of soil topped by a thin layer of peat (Kolari *et al*., 2022). Hyytiälä is part of the Integrated Carbon Observation System (ICOS) network, and it is a Station for Measuring Forest Ecosystem–Atmosphere Relations (SMEAR). Five Scots pine trees were sampled approximately once every 2 wk over 6 months of 2019, and current‐year needle (0N) sampling started when the sample size was large enough, from 12 June 2019 until 11 October 2019. We selected 1N sampling between 30/04 and 08/08, which gave more seasonal coverage of the growing season before 0N were large enough for sampling, and it allowed a 4‐wk sampling overlap for 0N and 1N. All needles were collected from sun‐exposed branches at 18 m tree height, between 13 and 16 h, and they were immediately transferred to paper bags in a cool box. They were promptly microwaved at 600 W for 1 min to stop enzymatic and metabolic activities (Wanek *et al*., 2001), and then oven‐dried for 24 h at 60°C. Needle samples were homogenized into a fine powder using FastPrep‐24™ (MP Biomedicals, Solon, OH, USA).

### Extraction and δ^2^H analysis of *n*‐alkanes

Long‐chain leaf wax *n*‐alkanes that are frequently used for palaeohydrological reconstructions (Liu *et al*., 2022) were extracted according to Wiesenberg & Gocke (2015). Homogenized needle material (0.3–1.5 g) was extracted using Soxhlet extraction with a mixture of dichloromethane : methanol (93 : 7; v/v). The extract weights were used to calculate the concentration of total lipid extracts (TLE). Extracts were then sequentially separated by solid phase extraction using KOH (5%)‐coated silica gel (SiO_2_, 60 Å) into neutral lipid‐, fatty acid‐ and polar lipid fractions. The *n*‐alkanes were separated from the neutral lipid fraction using solid phase extraction with activated SiO_2_ (100 Å, 110°C overnight). Squalene was added as an internal standard to the *n*‐alkane fraction before gas chromatographic (GC) analysis.

*n*‐Alkanes were identified using an Agilent (Santa Clara, CA, USA) 6890N GC equipped with a split/splitless injector coupled to an Agilent 5973 mass selective (MS) detector and external standards and quantified on a GC (Agilent 7890B) equipped with a multimode inlet (MMI) and flame ionization detector. Compound identification of *n*‐alkanes was performed using NIST/Wiley spectral libraries and comparison of retention times and mass spectra to external standards (Supelco 68281 with C_10_–C_40_ *n*‐alkanes). Both GC instruments were equipped with a J&W DB‐5MS narrow‐bore capillary column (50 m × 0.2 mm, 0.33 μm film thickness) and a deactivated precolumn (1.5 m). Helium was used as the carrier gas. The GC oven temperature increased from 70°C (held for 4 min) to 320°C (held for 20 min) at a rate of 5°C min^−1^. The MMI was kept at 80°C for 0.5 min, ramped to 450°C at a rate of 800°C min^−1^ (held for 2 min) and cooled to 250°C until the end of the oven program.

The *n*‐alkane hydrogen isotope composition was determined using a Thermo Fisher Scientific (Waltham, MA, USA) Trace 1320 GC equipped with an split/splitless (S/SL) injector connected via GC‐IsoLink II to a ConFlo IV and Delta V Plus isotope mass spectrometer (irMS). Injection was performed, in splitless mode, using an robotic sample handling (RSH) automated liquid sample handling tool using 1–3 μl with a 10‐μl syringe at least in triplicate, and in the case of low concentrations or too high deviation between replicates, even more often. For samples with very low alkane abundances, manual injections were performed, if necessary. The temperature of the S/SL injector was 280°C. The capillary column setup and the oven temperature program were identical to the oven temperature program used for quantification. All measurements were performed at least in triplicate. Calibration of δ^2^H_alkane_ values was done against A7 and B5 *n*‐alkane standard mixtures provided by the Schimmelmann Research laboratory (https://hcnisotopes.earth.indiana.edu/reference‐materials/materials‐descriptions/n‐alkanes.html, accessed 4 May 2024). Regular measurements of secondary standard mixtures (with *n*‐C_20_, *n*‐C_24_, *n*‐C_30_, *n*‐C_32_ alkanes) enabled determination of instrument performance and recalibration, if necessary. The precision of δ^2^H values of the standard mixtures was better than 1‰ for the individual compounds. If the measured values were outside this range, a reconditioning of the reactor or a recalibration was performed. The measurements of individual samples were run at least in triplicate and only accepted, if the precision of δ^2^H values was better than 2‰ for individual compounds. Otherwise, additional replicate measurements were conducted, or further measures were taken, such as adjustment of concentrations to receive satisfying results. The reported δ^2^H_alkane_ is provided as the weighted average of the most abundant *n*‐alkanes (*n*‐C_25_, *n*‐C_27_, and *n*‐C_29_).

### Extraction and δ^2^H analysis of WSC and starch

Water‐soluble carbohydrates were extracted from needle powder using the hot water extraction method, following the extraction procedure outlined by Wanek *et al*. (2001) and the purification procedure outlined by Rinne *et al*. (2012). Needle starch was extracted from the remaining pellet by enzymatic hydrolysis, according to Wanek *et al*. (2001) and Lehmann *et al*. (2019). These procedures are the same as those in Tang *et al*. (2023) (Supporting Information Methods S1).

Isotope analysis of WSC and starch followed Schuler *et al*. (2022). Samples were double packed into 5.5 × 9 mm silver foil capsules in duplicates. They were then offline equilibrated for 2 h then dried for a further 2 h in N_2_ gas, at 130°C (Schuler *et al*., 2022). Immediately afterwards, samples were transferred to a high‐temperature elemental analyzer system coupled to a Delta^Plus^ XP IRMS (Finnigan, Thermo Fisher Scientific) for ^2^H/^1^H measurement. Each sample was analyzed twice, and each round of analysis had an isotopically distinct water for the equilibration. The results typically show that 34–38% of H in sugars is exchangeable, depending on the type of sugar (Schuler *et al*., 2022). This was considered in the calculation for the nonexchangeable hydrogen isotope ratio (eqn 3 in Schuler *et al*., 2022). Results were normalized with in‐house sugar standards to the Vienna Standard Mean Ocean Water scale. For starch samples, 3 1N sampling days (in May) and 1 0N sampling day were estimated with only one isotopically distinct water; however, their data were included because their variability did not supersede overall seasonal trends, which were confirmed to have not been affected by small sample size. We corrected δ^2^H_starch_ and starch concentrations for α‐amylase by its relative weight in samples and its measured δ^2^H, which had a negligible (< 5‰) influence to 1N δ^2^H_starch_ and a relatively low maximum influence (< 13‰) to 0N δ^2^H_starch_ considering that the 0N δ^2^H_starch_ range was 100‰.

### Independent variables for time‐integrated comparisons to δ^2^H in organic compounds

Our selection of independent variables included a suite of environmental and physiological variables (Table 2). We downloaded RH, transpiration (*E*), net CO_2_ influx (*A*_n_), air temperature and air pressure data from the Smart SMEAR AVAA portal (Aalto *et al*., 2023), at half‐hourly resolution. RH was measured onsite by a Rotronic MP102H RH/T sensor at 16.8 m. *E* and *A*_n_ were measured using two automated, box‐shaped shoot chamber systems made of acrylic plastic (2.1 dm^3^), surrounding debudded shoots in the uppermost canopy (20 m; Aalto *et al*., 2014). Cuvettes were ventilated and equipped with a fan. *E* was calculated by applying a nonlinear equation to chamber H_2_O vapor concentrations during the first 5–35 s of intermittent chamber closures (Kolari *et al*., 2012), while *A*_n_ was represented by net needle CO_2_ influx, filtered to only include measurements above 0.1 μg m^−2^ s^−1^ to reduce bias from instrument error. When there were missing *E* or *A*_n_ data, we gap filled measurements by implementing the photosynthesis model by Leppä *et al*. (2022), which was parameterized to one of the cuvettes at the study site and represented the chamber's 2019 measurements well (*R* ^2^ = 0.86, RMSE = 0.04; Fig. S1 inset). We then calculated total leaf conductance to water vapor (*g*_t_; mol m^−2^ s^−1^) from *E* (mol m^−2^ s^−1^) using the following equation:

$$g_{t}=\frac{E\times \left(p-\dot{e}\right)}{\left(e_{i}-e_{a}\right)}$$

where p is air pressure (kPa), *e*_i_ is leaf intercellular vapor pressure (kPa), which we assumed to be saturated for the coarse‐scale implementation of *g*_t_ in this study, *e*_a_ is atmospheric vapor pressure (kPa) and $\dot{e}$ is $\left(e_{i}+e_{a}\right)/2$ (Gaastra, 1959; von Caemmerer & Farquhar, 1981; Cernusak *et al*., 2024). Sensitivity tests confirmed that, for the reported range of boundary layer conductance in *Pinus* species (2–2.84 mol m^−2^ s^−1^; Cernusak *et al*., 2022), the $g_{s}$ of trees in our study were low enough to largely control $g_{t}$ variability; therefore, $g_{t}$ was a suitable proxy for $g_{s}$. For measured δ^2^H_vapor_, δ^2^H_source_, δ^2^H_n‐water_ and ∆^2^H_n‐water_, we used data from Angove *et al*. (2023), where δ^2^H_source_ was represented by twig water δ^2^H. Since the time‐integrated analyses required continuous δ^2^H data throughout the growing season, we additionally modeled δ^2^H_source_, δ^2^H_vapor_, δ^2^H_n‐water_ and ∆^2^H_n‐water_ in Python (van Rossum & Drake, 2009; Jupyter, 2022). This involved a mass‐balanced‐based model of water isotopes in the rooting zone for δ^2^H_source_, and leaf water heavy isotope enrichment modeling using the Craig–Gordon model and its different corrections (Péclet, two‐pool and nonsteady state) for δ^2^H_n‐water_ and ∆^2^H_n‐water_ (Methods S2; Figs S2–S4). Model inputs included data from the ICOS carbon portal (Mammarella *et al*., 2024) and IsoGSM, an isotope‐enabled atmospheric circulation model (Yoshimura *et al*., 2008, 2011). We also explored how accurately we could infer δ^2^H_n‐water_ from measured δ^2^H_alkane_ in 1N and 0N. For this, we used the mean (−156‰), minimum (−133‰) and maximum (−192‰) isotope fractionation between δ^2^H_n‐water_ and δ^2^H_alkane_ (ε_bio_) reported by Hepp *et al*. (2021).

**Table 2:** Independent factors selected for comparison to nonstructural carbohydrate δ^2^H and *n*‐alkane δ^2^H, in needles.

Factor	Definition	Reason
RH	Atmospheric relative humidity (%)	Related to leaf water δ^2^H and δ^18^O (Cernusak *et al*., 2016)
*E*	Transpiration rate (mol m^−2^ s^−1^)	Relates to leaf water δ^2^H and δ^18^O via the Péclet effective path length (Song *et al*., 2013). The usefulness of the Péclet correction for tree‐ring δ^18^O is under question (Ogée *et al*., 2009; Barbour *et al*., 2024); thus, it is valuable to examine for carbohydrate δ^2^H
*A*_n_	Net assimilation rate, represented by net CO_2_ influx (μmol m^−2^ s^−1^) measured by cuvette gas exchange, when influx measurements > 0.1 μg m^−2^ s^−1^	Represents assimilation rate and thus autotrophic status (Yakir, 1992). Related to the hydrogen isotope offset between WSC and water in a controlled experiment (Lehmann *et al*., 2024a).
*g*_s_	Stomatal conductance (mol m^−2^ s^−1^)	Related to photosynthetic activity (Wong *et al*., 1979) and used to model δ^2^H_n‐water_ (e.g. Cernusak *et al*., 2022). Observed relationship to the hydrogen isotope offset between WSC and water in a controlled experiment (Lehmann *et al*., 2024a).
δ^2^H_n__‐water_	Modeled bulk needle water ^2^H ratio to ^1^H. Values relative to VSMOW.	A source of ^2^H to *n*‐alkanes and carbohydrates (Cormier *et al*., 2018), and *n*‐alkanes are hypothesized to represent δ^2^H_n‐water_ (Tipple *et al*., 2015; Zech *et al*., 2015; Hepp *et al*., 2021)
Δ^2^H_n__‐water_	Modeled needle water ^2^H enrichment above modeled source water	Represents variability of water more exposed to Δ^2^H_n‐water_, which could be more representative of photosynthetic water by leaf water heterogeneity (Liu *et al*., 2021; Baca Cabrera *et al*., 2023)
δ^2^H_source_	Modeled source water ^2^H ratio to ^1^H. Values relative to VSMOW.	Interpreted from *n*‐alkane δ^2^H in palaeohydrological studies (Freimuth *et al*., 2017)

VSMOW, Vienna Standard Mean Ocean Water; WSC, water‐soluble carbohydrates.

### Data analyses

**Fig. 1:**
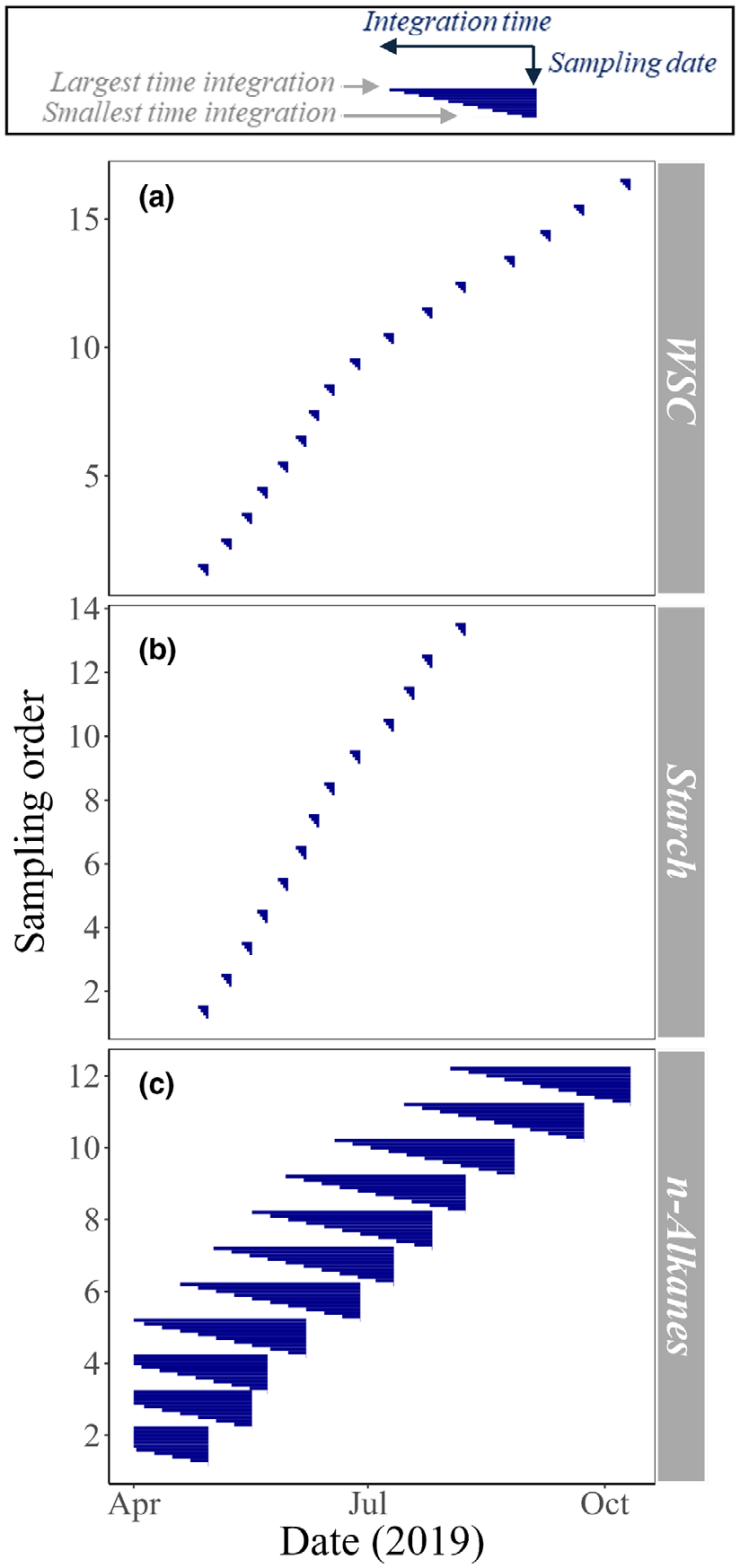
Each sampling event, in chronological order (Sampling order), and the range of dates included in time‐integrated analyses; for water‐soluble carbohydrates (WSC) (a), starch (b) and *n*‐alkanes (c), in *Pinus sylvestris* needles at Hyytiälä Forest.

Data analysis was conducted in R (v.4.2.1; R Core Team, 2022). To explore how our δ^2^H_alkane_ data aligns with previous studies, we inspected temporal trends in δ^2^H values in all measured compounds and mixtures, and modeled δ^2^H_n__‐water_ and δ^2^H_source_. We then prepared time‐integrated means of independent variables (Table 2). Data were filtered between 09:00 and 15:00 h, then averaged, and time integration was implemented during averaging. One day for time integration was represented by the mean of the sampling day, while a 2‐d integration time was represented by an average of the sampling day and its preceding day. This was repeated for up to five integration days for δ^2^H_WSC_ and δ^2^H_starch_ (Fig. 1a,b), based on the WSC δ^18^O integration time used for the same site (Leppä *et al*., 2022), during the time window that coincided with our sampling period. For δ^2^H_alkane_, we used a period of 0–10 integration weeks to enhance the maximum time integration coverage and minimize exposure to uncertainties from the dormant winter period preceding the growing season (Fig. 1c). The starting date for modeled δ^2^H_source_, δ^2^H_n‐water_ and ∆^2^H_n‐water_ was 01 April 2019 because earlier starting dates were not in the scope of model validation since there is uncertainty in estimating winter tree water uptake for nonsteady state δ^2^H_source_ predictions. Resultantly, the first three 1N sampling events had maximum time integrations of 4–5, 6–7 and 7–8 wk, respectively.

After integration times had been applied to selected independent variables (Table 2), they were compared with δ^2^H_WSC_, δ^2^H_starch_ and δ^2^H_alkane_ using Spearman's rank correlations. A Holm correction was applied to *P*‐values for multiple integration day comparisons. The correlation results were visualized using an annotated color heat map, following the approach by Tang *et al*. (2024). We selected the independent factors, and their integration times, which were most strongly correlated with δ^2^H_WSC_ and δ^2^H_alkane_ for focused inspection by linear mixed models (LMMs). Since 1N δ^2^H_alkane_ was related to multiple factors with negligible differences in rank relationship (ρ < 0.04), the focused LMM inspection featured the three factors most relevant to palaeohydrological reconstructions. Tree identity and sampling date were random intercept factors for δ^2^H_WSC_ LMMs, but more parsimony was needed to validate an LMM for δ^2^H_alkane_; therefore, its random intercept was tree identity without sampling date. An 0N sampling event in October was not included in the LMM because the short integration period of δ^2^H_WSC_ (Fig. 1a) was susceptible to bias from the October sampling event when the growing season had clearly ended.

## Results

### Concentrations of total lipid extracts and carbohydrates

**Fig. 2:**
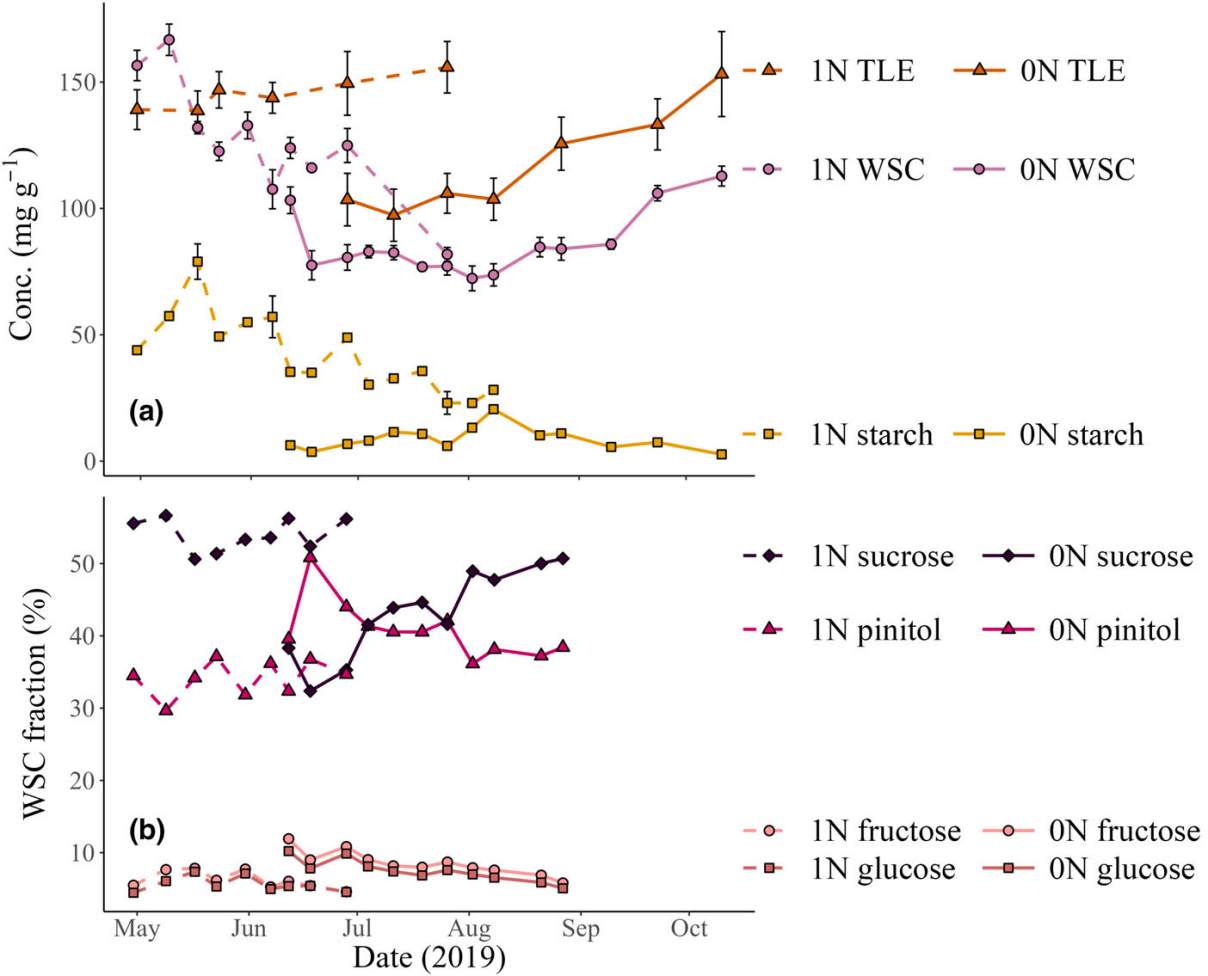
Time series of concentration and composition changes in carbohydrates and total lipid extracts (TLE). (a) Concentration of TLE, water‐soluble carbohydrates (WSC) and starch, in 1‐yr‐old needles (1N) and current‐year needles (0N), during their respective sampling periods. Error bars represent SE, which was not always available for starch because its low concentrations required sample pooling. (b) Percentage contribution of different WSC constituents to the bulk WSC pool in 1N and 0N. Data from five *Pinus sylvestris* trees at Hyytiälä Forest, central Finland.

Concentrations of WSCs, starch and TLE were typically higher in 1N than in 0N (Fig. 2a). There was a decline in WSC concentrations of 1N during the sampling period, from *c*. 160 to *c*. 80 mg g^−1^. Meanwhile, in 0N, there was a small (*c*. 25 mg g^−1^) decrease in WSC concentrations at the start of the sampling period, which then stabilized, and gradually increased between September and October, to *c*. 110 mg g^−1^. Mean concentrations of TLE in 1N were relatively stable, varying between 139 and 156 mg g^−1^. Meanwhile, in 0N, TLE concentration gradually increased from 100 to 150 mg g^−1^ to eventually reach equivalent concentrations to 1N, by the end of the sampling period. Starch concentrations in 1N peaked during mid‐May. In 0N, starch concentrations were low (*c*. 3–20 mg g^−1^) compared with all other measured concentrations, and they peaked in August (Fig. 2a).

The weight percentage of pinitol (a sugar alcohol) fraction in 1N WSC was rather invariable, ranging between 30% and 37% (Fig. 2b). Meanwhile, the 0N pinitol fraction declined from 51% to 36% during its sampling period, alongside decreases of 5% and 6% in glucose and fructose, respectively. These declines corresponded with an increase in the sucrose fraction from 30% to 51%.

### Temporal trends in δ^2^H

**Fig. 3:**
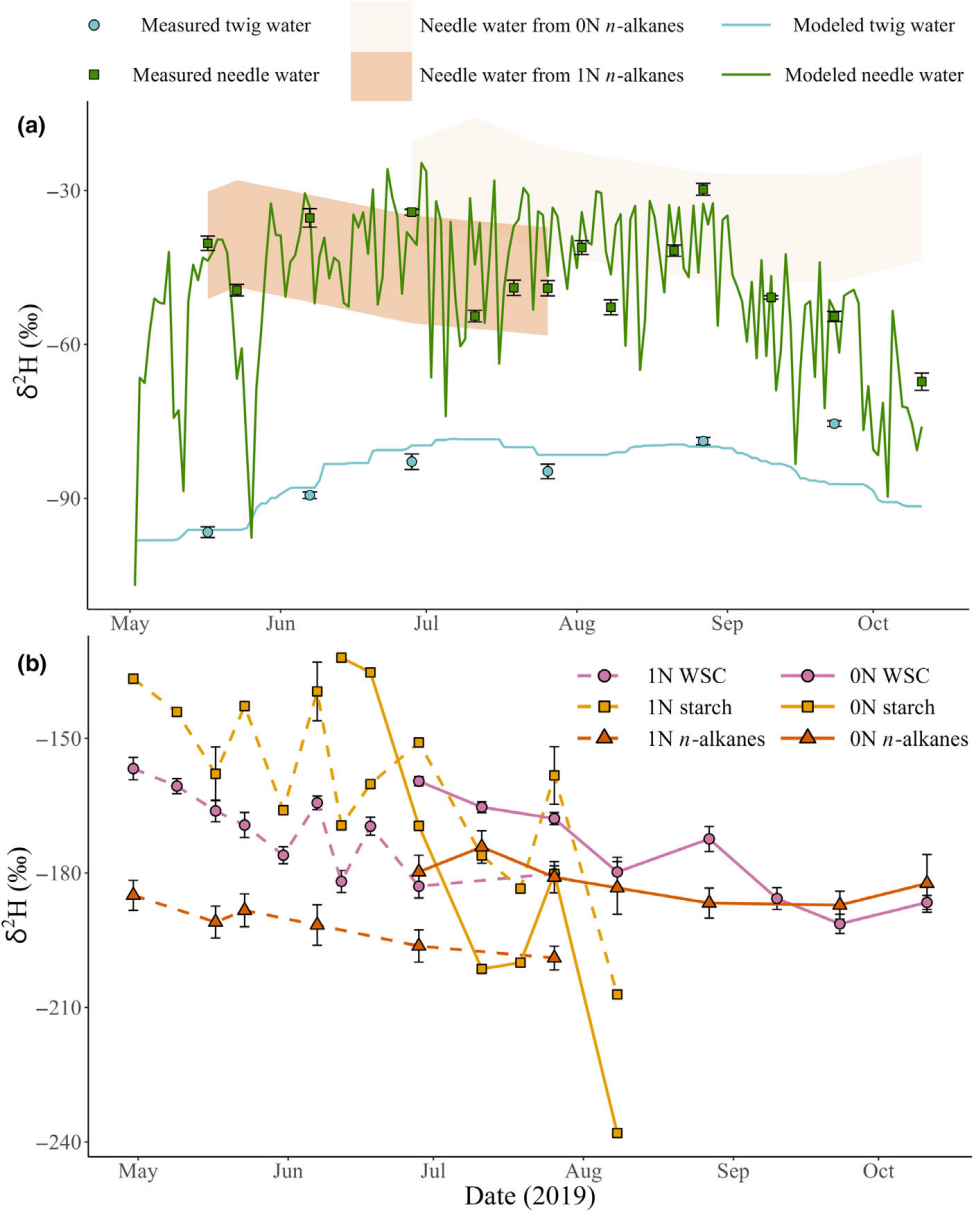
Temporal variability of δ^2^H in different compounds and mixtures from 1‐yr‐old (1N) and current‐year (0N) needles of five *Pinus sylvestris* trees during the 2019 growing season at Hyytiälä forest, central Finland. (a) Measured δ^2^H of 1‐yr‐old needle water (green datapoints; 1N δ^2^H_n‐water_) with its modeled values (green line; Craig–Gordon model with Péclet correction), and measured twig water δ^2^H (blue datapoints; δ^2^H_source_), with the modeled values used for gap filling (blue line; mass balanced‐based model of the rooting zone). Shaded areas (orange) show δ^2^H_n‐water_ when inferred from *n*‐alkane δ^2^H (δ^2^H_alkane_) using the mean (−156‰) and minimum (−133‰) isotope fractionation between δ^2^H_n‐water_ and δ^2^H_alkane_ (ɛ_bio_) reported by Hepp *et al*. (2021). (b) δ^2^H of water‐soluble carbohydrates (WSC, δ^2^H_WSC_), starch (δ^2^H_starch_) and *n*‐alkanes in 1‐yr‐old needles (1N; dashed lines) and current‐year needles (0N; solid lines). Error bars show SE, which was not always available for δ^2^H_starch_ because of sample pooling, to accommodate low starch concentrations.

Needle gas exchange had a strong role in measured and modeled needle water δ^2^H (δ^2^H_n__‐water_), as both exhibited distinctly different and larger variability compared to measured and modeled twig water δ^2^H (δ^2^H_source_; Fig. 3a). In late September, δ^2^H_source_ was underestimated, as reported for δ^18^O_source_ (Leppä *et al*., 2022). Nonetheless, it did not bias modeled δ^2^H_n‐water_ (Fig. 3a). When measured δ^2^H_source_ and δ^2^H_vapor_ were missing, the predictions for δ^2^H_n‐water_ were moderately accurate (Fig. S5; Table S1). This was substantial accuracy for this study to implement multiday or multiweek time‐integrated comparisons to organic δ^2^H variability (Fig. 3). When exploring δ^2^H_n‐water_ predictions from δ^2^H_alkane_ using mean (−156‰) to minimum (−133‰) reported ɛ_bio_ values, δ^2^H_n‐water_ from 1N δ^2^H_alkane_ was, at a low resolution, within the general range and direction of measured 1N δ^2^H_n‐water_ (Fig. 3a). But δ^2^H_n‐water_ from 0N δ^2^H_alkane_ did not capture the general variability observed in measured 1N δ^2^H_n‐water_. δ^2^H_n‐water_ predictions from the maximum reported ɛ_bio_ value (−192‰), not shown, were highly unrealistic; at *c*. −6 to 6 from 1N δ^2^H_alkane_ and 4–15‰ from 0N δ^2^H_alkane_.

When 1N δ^2^H_WSC_ overlapped with 0N δ^2^H_WSC_ measurements, δ^2^H_WSC_ was lower in 1N than in 0N (Fig. 3b). In 1N, δ^2^H_WSC_ was significantly (18‰; *P* < 0.01; *t* = −3.94; *n* = 10) lower than δ^2^H_starch_, on sampling days when they were both analyzed. However, this was not reciprocated in the few pairwise measurements available for 0N (*P* > 0.05; *t* = 1.86; *n* = 4). δ^2^H_starch_ in 0N, the most variable of all organic compound pools (Table 3), began with high values (δ^2^H ≈ −132‰) close to the mean branch phloem δ^2^H_WSC_ (−137.5‰) at the start of 0N sampling, followed by a steep decrease to *c*. −238‰, interspersed by a relatively small increase during July. In 1N, the δ^2^H_starch_ and δ^2^H_WSC_ both generally decreased during the growing season, sharing some peaks and troughs at similar sampling points, albeit more subtly for δ^2^H_WSC_. Similarly, δ^2^H_alkane_ gradually decreased in 1N, from −185‰ to −199‰.

**Table 3:** Means (±SE) and interquartile ranges of δ^2^H (‰) in different compounds and mixtures.

	Twig	Current‐year needles	One‐year‐old needles	Branch phloem
Mean ± SE				
Water	−84.8 ± 1.3	–	−45.1 ± 1.4	–
WSC	–	−176.1 ± 1.9	−170.6 ± 1.4	−137.5 ± 2.3
Starch	–	−179.5 ± 14.3	−155.6 ± 3.6	−73.7 ± 2.7
*n*‐Alkanes	–	−182.4 ± 1.6	−192.5 ± 1.5	–
Interquartile range				
Water	11	–	15.7	–
WSC	–	21.2	14.6	12.7
Starch	–	48.3	24.3	4.4
*n*‐Alkanes	–	11	11.5	–

Data from five *Pinus sylvestris* trees monitored during 2019, at Hyytiälä Forest, central Finland. WSCs, water‐soluble carbohydrates.

Unlike for δ^2^H_WSC_ and δ^2^H_starch_, the interquartile range for δ^2^H_alkane_ had a negligible difference between needle ages (0.5‰), and its interquartile range was as low as the less variable range of δ^2^H_source_ (11‰). On average, δ^2^H_alkane_ was the most depleted of all measured organic compounds and mixtures (Fig. 3b; Table 3). Notably, it was not lower than δ^2^H_WSC_ in 0N at the end of the growing season (Fig. 3b).

Mean isotope fractionation between modeled δ^2^H_n‐water_ and leaf organic compound δ^2^H (following eqn 4 in Holloway‐Phillips *et al*., 2022) is given in Table S2, but these are approximate estimates because of the variable integration times of different organic compounds.

### Nonstructural carbohydrate δ^2^H relationships to time‐integrated factors

**Fig. 4:**
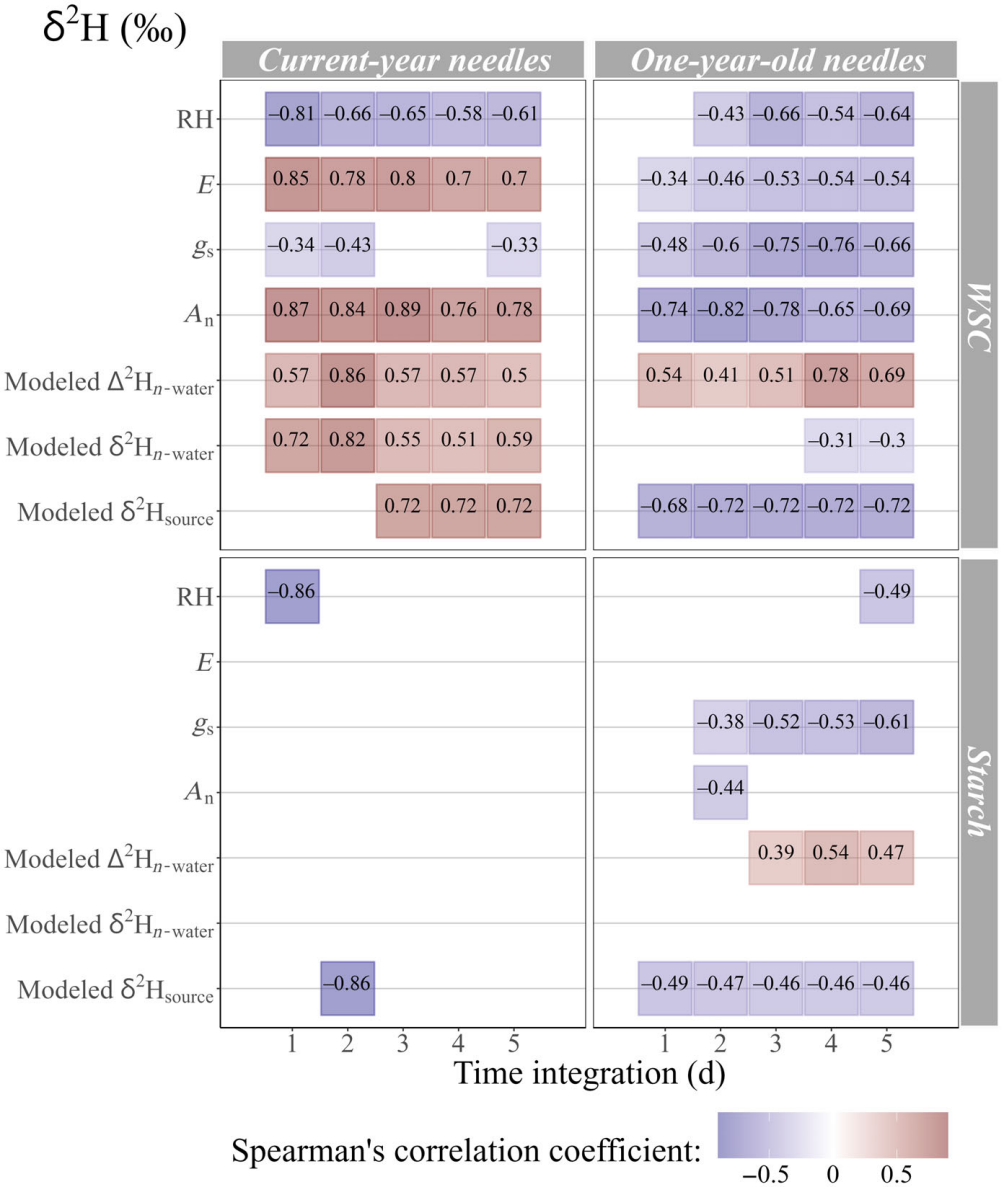
Time‐integrated Spearman's correlations identifying physiological and environmental signals in nonstructural carbohydrate δ^2^H (water‐soluble carbohydrate δ^2^H (WSC) and starch δ^2^H). Up to 5 d of time integration are used, based on evidence from WSC δ^18^O for the specific spatiotemporal context of this study (Leppä *et al*., 2022). Physiological factors include the following: transpiration rate (*E*, mol m^−2^ s^−1^), stomatal conductance (*g*_s_, mol m^−2^ s^−1^) and net assimilation rate (*A*_n_, μmol m^−2^ s^−1^). Environmental factors include the following: relative humidity (RH, %), modeled needle water ^2^H enrichment (∆^2^H_n‐water_, ‰), modeled needle water δ^2^H (δ^2^H_n‐water_, ‰) and modeled source water δ^2^H (δ^2^H_source_, ‰). Isotope measurements are from current‐year needles (0N) and 1‐yr‐old needles (1N) of five *Pinus sylvestris* trees monitored throughout the 2019 growth season, while gas exchange data are from continuous mature needle cuvette measurements at half‐hourly resolution, at Hyytiälä Forest, central Finland.

The Spearman's correlations between δ^2^H and selected factors (Table 2) varied between needle generation, and between WSC and starch (Fig. 4). In both 1‐yr‐old needles (1N) and current‐year needles (0N), δ^2^H_WSC_ was most strongly correlated with *A*_n_ (*P* < 0.05). However, the trends were opposite: 0N was positively related to *A*_n_ (ρ = 0.89), while 1N was negatively correlated with *A*_n_ (ρ = −0.82). The optimal integration time for both needle ages was 2–3 d, and both days had strong correlations for both needle ages. In addition to the strong correlations between δ^2^H_WSC_ and time‐integrated *A*_n_, δ^2^H_WSC_ was, to a lesser extent, significantly related to modeled 1N δ^2^H_n‐water_ and ∆^2^H_n‐water_ in both needle ages. Just as 1N δ^2^H_WSC_ was negatively related to time‐integrated *A*_n_ while 0N δ^2^H_WSC_ was positively related to time‐integrated *A*_n_, 1N δ^2^H_WSC_ had a negative relationship with modeled 1N δ^2^H_n‐water_ while 0N δ^2^H_WSC_ had a positive relationship. δ^2^H_WSC_ was most strongly related to modeled 1N δ^2^H_n‐water_ after four integration days for 1N (ρ = −0.31), and 2 d for 0N (ρ = 0.82). Meanwhile, δ^2^H_WSC_ was positively correlated with modeled 1N ∆^2^H_n__‐water_ in both needle ages, and the strongest correlations were after 4 d for 1N (ρ = 0.78), and 2 d for 0N (ρ = 0.86). Exclusively for 0N, the strongest signals in δ^2^H_WSC_ were closely followed by RH and *E,* which were strongest on the day of sampling (Fig. 4).

δ^2^H_starch_ in 0N was most strongly correlated with δ^2^H_source_ after a 2‐d integration (ρ = −0.86) and RH on the sampling day (ρ = −0.86; Fig. 4). By contrast, in 1N, the strongest relationship was a weak correlation with *g*_s_ after five integration days (ρ = −0.61, *P* < 0.05).

**Fig. 5:**
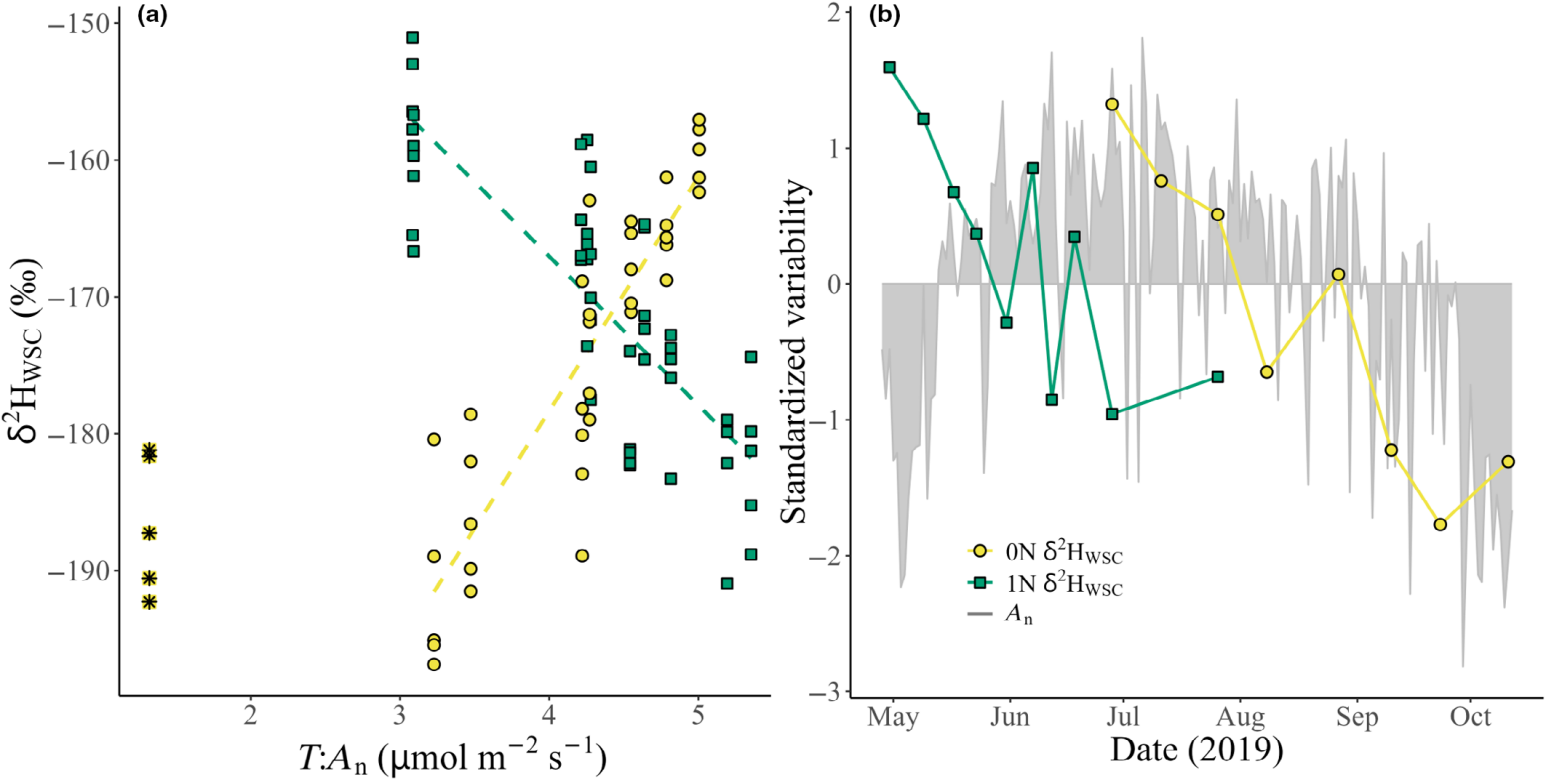
Water‐soluble carbohydrate (WSC) δ^2^H (δ^2^H_WSC_) relationships to net assimilation rate (*A*_n_, μmol m^−2^ s^−1^), for current‐year needles (0N, yellow series) and 1‐yr‐old needles (1N, green series) from five *Pinus sylvestris* trees at Hyytiälä Forest, central Finland. *A*_n_ data are from continuous 1N needle cuvette measurements at half‐hourly resolution. In (a), *A*_n_ is time integrated (*T*:*A*_n_) to 3 d for 0N (*R* ^2^(M) = 0.8, *P* < 0.05) and 2 d for 1N (*R* ^2^(M) = 0.66, *P* < 0.05), dashed lines show significant linear mixed model fits, and asterisks show an 0N δ^2^H_WSC_ outlier from a sampling day in October, when the growing season had ended. In (b), *A*_n_ is not time‐integrated, and each of the variables were standardized (centered (mean of a variable subtracted from its values) and then scaled (divided by the SD, for each variable)).

Linear mixed models confirmed a strong positive relationship between time‐integrated *A*_n_ (*T*:*A*_n_) and 0N δ^2^H_WSC_ within the growing season (Table 4; Fig. 5a). By contrast, for 1N, the relationship was weaker and negative. Although δ^2^H_WSC_ decreased in both 0N and 1N throughout the growing season, *A*_n_ increased in 1N while δ^2^H_WSC_ decreased (Fig. 5b). Meanwhile, in 0N, the decrease in *A*_n_ closely aligned with the decrease in δ^2^H_WSC_ toward the end of the growing season (Fig. 5b).

**Table 4:** Linear mixed model fits for time‐integrated signals (net assimilation rate, *T*:*A*_n_, μmol m^−2^ s^−1^; modeled needle water δ^2^H, *T*:δ^2^H_n‐water_, ‰; gap filled source water δ^2^H, *T*:δ^2^H_source_, ‰) in water‐soluble carbohydrate δ^2^H (δ^2^H_WSC_) and *n*‐alkane δ^2^H (δ^2^H_alkane_), in current‐year needles (0N) and 1‐yr‐old needles (1N) from five *Pinus sylvestris* trees at Hyytiälä Forest, central Finland.

Regressor	Response	Intercept	Slope	ICC	*R* ^2^(M)	*R* ^2^(C)
3‐d *T*:*A*_n_ 2‐d *T*:*A*_n_	0N δ^2^H_WSC_	−246.78 ± 6.56	**17.11** ± 1.49	0.13	0.8	0.93
	1N δ^2^H_WSC_	−123.52 ± 7.29	**−10.88** ± 1.64	0.18	0.66	0.84
10‐wk *T*:RH	1N δ^2^H_alkane_	−134.38 ± 9.17	**−1.13** ± 0.17	0.47	0.31	0.77
5‐wk *T*:δ^2^H_n‐water_		−234.93 ± 7.42	**−0.85** ± 0.14	0.47	0.29	0.75
5‐wk *T*:δ^2^H_source_		−246.02 ± 9.12	**−0.59** ± 0.1	0.46	0.29	0.75

Bold when significant (P < 0.05). ICC, intraclass correlation; *R* ^2^(C), conditional *R* ^2^; *R* ^2^(M), marginal *R* ^2^; RH, relative humidity; WSC, water‐soluble carbohydrates.

### *n*‐Alkane δ^2^H relationships to time‐integrated factors

**Fig. 6:**
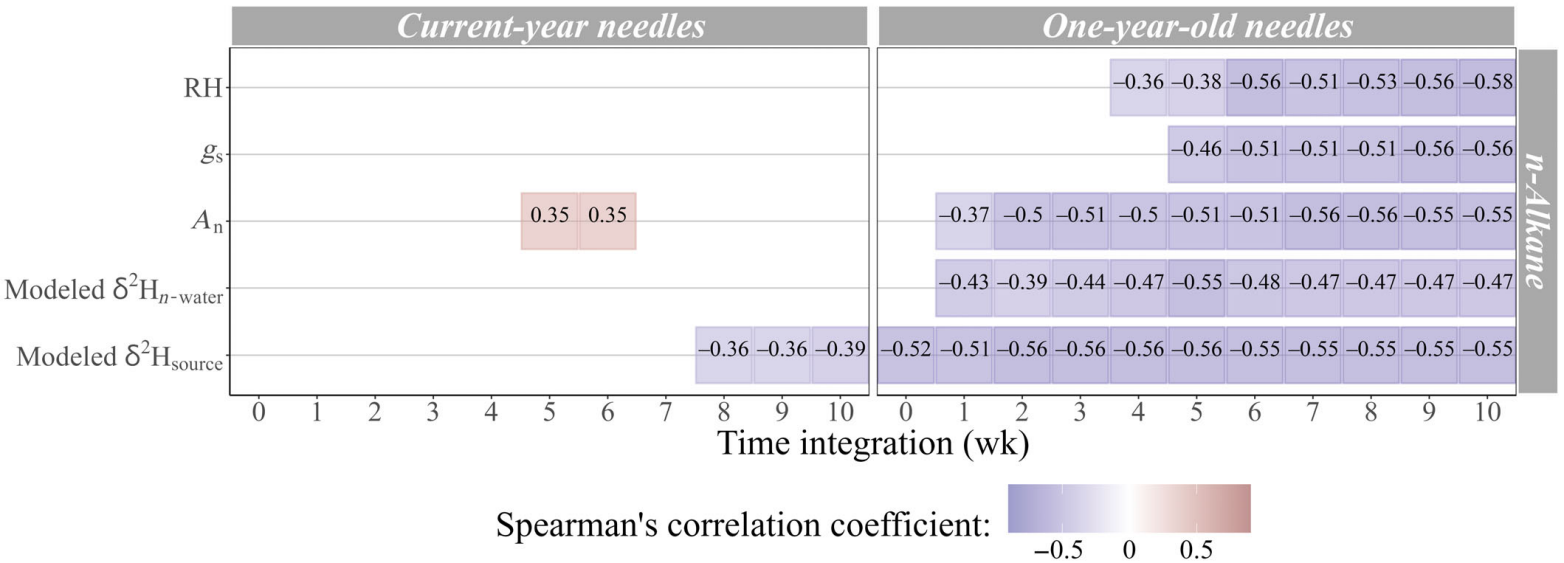
Significant time‐integrated Spearman's correlations between *n*‐alkane δ^2^H and the following factors: relative humidity (RH) (%), stomatal conductance (*g*_s_, mol m^−2^ s^−1^), net assimilation rate (*A*_n_, μmol m^−2^ s^−1^), modeled needle water δ^2^H (δ^2^H_n‐water_, ‰) and modeled source water δ^2^H (δ^2^H_source_, ‰). *n*‐Alkane δ^2^H is from current‐year needles (0N) and 1‐yr‐old needles (1N) of five *Pinus sylvestris* trees monitored during 2019 at Hyytiälä Forest, central Finland. Gas exchange data are from continuous mature needle cuvette measurements at half‐hourly resolution.

**Fig. 7:**
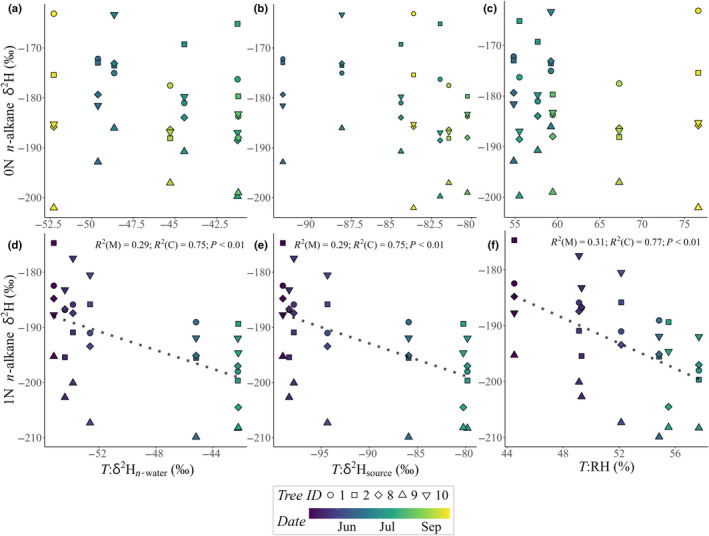
Needle *n*‐alkane δ^2^H (‰) relationships to time‐integrated δ^2^H in water pools and relative humidity (RH), to examine hydrological signals in *n*‐alkane δ^2^H when at their strongest. Current‐year needle (0N) *n*‐alkane δ^2^H relationships to 10‐wk integrated (a) modeled needle water δ^2^H (*T*:δ^2^H_n‐water_, ‰), (b) gapfilled source water δ^2^H (*T*:δ^2^H_source_, ‰) and (c) atmospheric relative humidity (*T*:RH, %). One‐year‐old needle (1N) *n*‐alkane δ^2^H relationships to 5‐wk integrated (d) *T*:δ^2^H_n‐water_ (‰), (e) *T*:δ^2^H_source_ (‰) and (f) 10‐wk integrated *T*:RH (%), with dashed lines showing significant linear mixed model fits. Data are from five *Pinus sylvestris* trees at Hyytiälä Forest, central Finland, during 2019.

0N δ^2^H_alkane_ was not significantly correlated with modeled δ^2^H_n‐water_ at any time integration (*P* > 0.05; Fig. 6). Instead, it had a weak negative relationship to modeled δ^2^H_source_ that became stronger as the time integration increased to 10 wk (ρ = −0.39, *P* < 0.05). It was also weakly correlated with *A*_n_, after 5 and 6 integration weeks (ρ = 0.35, *P* < 0.05). The weak relationship between 0N δ^2^H_alkane_ and δ^2^H_source_ after 10 wk of integration (Figs 6, 7b) did not fulfill the model validation requirements for a generalized LLM. There were noticeable intertree differences in δ^2^H_alkane_, for both needle ages, indicated by the relatively large vertical scatter in Fig. 7.

1N δ^2^H_alkane_ exhibited stronger correlations to tested factors compared to 0N δ^2^H_alkane_ (Fig. 6). It was correlated with a subselection of the factors tested (Table 2). The prevailing signals in 1N δ^2^H_alkane_ were RH, δ^2^H_source_, δ^2^H_n‐water_, *A*_n_ and *g*_s_, and their rank (ρ) differences were < 0.04 (Fig. 6). In most cases, the strongest signals were observed between 5 and 10 integration weeks, except that δ^2^H_source_ exhibited an invariable (ρ < 0.02) signal between 2 and 10 wk. The LLM that compared 1N δ^2^H_alkane_ with hydrological factors (*T*:δ^2^H_n‐water_, *T*:δ^2^H_source_, RH) identified a high likelihood (46–47%) that δ^2^H_alkane_ from the same tree would correlate (Table 4). Excluding the role of tree identity in the relationship between 1N δ^2^H_alkane_ and *T*:δ^2^H_n‐water_ (random intercept model), there was a weak (*R* ^2^(M) = 0.29) negative relationship; a trend that was mirrored by the relationship between 1N δ^2^H_alkane_ and *T*:δ^2^H_source_ albeit with a milder gradient (Fig. 7d,e; Table 4). The relationship was marginally stronger (*R* ^2^(M) = 0.31) for RH (Fig. 7f; Table 4).

## Discussion

Our study is the first to investigate time‐integrated, physiological and environmental signals in δ^2^H of *n*‐alkanes and carbohydrates in a natural forest during a growing season. This was feasible from an intensive field survey of a highly monitored site (ICOS, SMEAR), supported by modeling for gap‐filling missing measured data. Nonetheless, our results cannot provide the causality achieved from a controlled glasshouse experiment. Instead, they contextualize the role of different factors influencing δ^2^H amidst multiple seasonally variable factors occurring at natural frequencies. We show that, in a natural boreal forest, prevailing intraseasonal physiological and environmental signals can be identified from δ^2^H_alkane_, δ^2^H_WSC_ and δ^2^H_starch_ after accounting for an integration time of days or weeks.

### *n*‐Alkane δ^2^H relationships to hydrological factors were regulated by physiological processes

0N δ^2^H_alkane_ did not have a substantial relationship with δ^2^H of any of the water pools investigated (Figs 6, 7). The strongest observed relationship was a weak Spearman's rank correlation (ρ = −0.39) to δ^2^H_source_ after 8–10 wk of time integration. This supports previous evidence that δ^2^H_alkane_ is strongly affected by complex biosynthetic ^2^H‐fractionation (Baan *et al*., 2023a). Furthermore, one of the two remaining factors related to 0N δ^2^H_alkane_, after *T*:δ^2^H_source_, was *T*:*A*_n_ (3 or 5 wk), which had a weak positive correlation (ρ = 0.35; Fig. 6). Its weak positive trend could have indicated a switch from heterotrophy to autotrophy (Tipple & Ehleringer, 2018), and the absence of a δ^2^H_n‐water_ signal might have been due to incomplete autotrophy (Zhu *et al*., 2020). Another potential reason is that gas‐exchange data were not available for 0N, and inaccuracies could have been introduced by using gas‐exchange data from older needle generations, but the multiweek averaging that covered large seasonal change, likely superseded gas‐exchange differences between needle ages (Fig. S1). The different origins of NADPH used for *n*‐alkane synthesis (Cormier *et al*., 2018; Wijker *et al*., 2019; Maloney *et al*., 2024), relating to biochemical fluxes, could have also contributed to the large intertree differences in δ^2^H_alkane_ in both 0N and 1N.

1N δ^2^H_alkane_ was related to a subselection of factors (RH, δ^2^H_source_, *A*_n_, *g*_s_ and δ^2^H_n‐water_), which had marginal differences in signal strength (Fig. 6). Relative humidity was marginally the strongest signal (Figs 6, 7), which could have stemmed from its indirect relationships to both δ^2^H_n‐water_ and δ^2^H_source_ (Cernusak *et al*., 2022; Methods S2). Interestingly, 1N δ^2^H_alkane_ were negatively related to δ^2^H_n‐water_ (Figs 6, 7), which suggests variability in biosynthetic isotope fractionation playing a significant role during the imprinting of hydrological signals to δ^2^H_alkane_ (Baan *et al*., 2023a). A potential explanation is that higher δ^2^H_n‐water_ indicated favorable evaporative conditions, which promoted the use of more chloroplast‐derived NADPH for wax synthesis, which is likely more ^2^H‐depleted than in cytosol‐derived NADPH (Sessions *et al*., 1999); more details on potential changes in lipid biosynthesis pathways can be found in Zhou *et al*. (2016). Furthermore, δ^2^H_alkane_ includes a δ^2^H signal from both chloroplastic water and cytosolic water – any variability in the contributions from these water pools could influence δ^2^H_alkane_ (Holloway‐Phillips *et al*., 2022). Since chloroplastic and cytosolic waters are assumed to comprise different proportions of water exposed to evaporative enrichment (Barbour & Farquhar, 2000; Cernusak *et al*., 2004; Holloway‐Phillips *et al*., 2022), changes in their relative contributions to δ^2^H_alkane_ would lead to fluctuations in δ^2^H_alkane_ between enriched and unenriched leaf water, perhaps partly reflected here as fluctuations between ∆^2^H_n‐water_ and δ^2^H_n‐water_. Nonetheless, since δ^2^H_alkane_ was negatively related to both δ^2^H_n‐water_ and δ^2^H_source_ (Figs 6, 7; Table 4), water compartmentation and heterogeneity are not likely as temporally influential to δ^2^H_alkane_ compared with other sources of variability, including sourcing NADPH and other precursor molecules from long‐term storage. Accordingly, it was interesting to observe *A*_n_ and *g*_s_ signals in 1N δ^2^H_alkane_, after time integration periods beyond 1 and 5 wk, respectively (Fig. 6).

Ultimately, it was not expected to find a relationship with δ^2^H_n‐water_ in 1N, because δ^2^H_alkane_ was supposedly from only the early stages of needle growth. However, Ofiti *et al*. (2023) showed that needles from different years of the conifer *Picea mariana* had the same *n*‐alkane concentration responses to a warming treatment. This suggests that *de novo* biosynthesis of *n*‐alkanes in mature needles might be substantial enough for needle δ^2^H_alkane_ to respond to environmental variability and be a more reliable indicator of processes correlating with δ^2^H_n‐water_. This could be a result of less 1N exposure to variability in biosynthetic fractionation (Sachse *et al*., 2015; Tipple & Ehleringer, 2018), such as variability in the synthesis pathway for NADPH‐derived H atoms (Zhou *et al*., 2016; Maloney *et al*., 2024). This could have been interlinked with a lower dependency on precursor molecules originating from long‐term storage, which additionally have different isotopic signals (Lehmann *et al*., 2024b). Since mature needles are generally those that senesce, this is a promising message for the interpretation of δ^2^H_alkane_, because this might represent processes related to a time‐integrated evaporative signal from the duration of the needle lifespan. Our data are in line with other studies observing a renewal of needle alkanes during their lifespan (Ofiti *et al*., 2023), similar to other plant types (Srivastava & Wiesenberg, 2018; Speckert *et al*., 2023). As the alkane concentrations do not increase during the lifetime of leaves, this argues for a release of these epicuticular compounds into the environment as aerosols (Conte & Weber, 2002). Likely, the majority of these alkanes enter the soil in the vicinity of their plants, thus contributing an alkane signal from presenescent leaves, capturing the time until they are released. However, there is scarce information on the renewal rates of alkanes in needles (Ofiti *et al*., 2023) and leaves (Speckert *et al*., 2023) of mature trees, which hampers a quantitative assessment of the contribution of senesced needles compared with living needles to the soil alkane pool. Until such information is available, we have to keep in mind that the soil alkane composition is a composite of the alkane signal that we measure on tree biomass, despite the promising results we received in the current study.

### Water‐soluble carbohydrate δ^2^H can have mixed relationships to *A*_n_

This is the first study to relate needle gas exchange to δ^2^H_WSC_ in a natural forest. The relationships between *T*:*A*_n_ and δ^2^H_WSC_ support findings from glasshouse experiments showing that leaf gas exchange, such as dark respiration and net photosynthesis, has a strong role in sugar δ^2^H (Holloway‐Phillips *et al*., 2022). We show that physiology, in this case *A*_n_, can be the predominant signal in needle δ^2^H_WSC_ in a natural boreal forest (Figs 4, 5). Gas‐exchange measurements were not available for 0N, and correlations for 0N were performed using needle gas exchange data from mature needles, which may have affected the outcomes. Nonetheless, the seasonal variability in *A*_n_ was likely much larger than needle generation differences (Figs 5b, S1), and there were relatively strong *E* and RH signals observed in 0N δ^2^H_WSC_ (Fig. 4). Overall, given that the 1N and 0N sampling periods cover different parts of the growing season, there are multiple potential mechanistic reasons for their differences. For example, the positive relationship between δ^2^H_WSC_ in 0N and *A*_n_ could be because they coincided with an increasing sugar pool size (Fig. 2a), and if it subsequently had a lower turnover rate, then the sugar pool could have been more exposed to ^2^H enrichment by H atom exchange with surrounding needle water (Holloway‐Phillips *et al*., 2022). The WSC pool is likely exposed for extended periods of time in a natural forest because our study, and Leppä *et al*. (2022) suggest that the WSC pool can be an accumulation from multiple days. To evaluate the role of turnover rate to δ^2^H_WSC_ in a natural forest, implementation of nonsteady state WSC isotope modeling is needed to determine the age of the 0N WSC pool, and its interactions with δ^2^H_WSC_. Another potential explanation for the relationship between *T*:*A*_n_ and 0N δ^2^H_WSC_ is the seasonal increase in the sucrose fraction of the WSC pool (Fig. 2b); if it was more ^2^H depleted, it could have contributed to the decreasing δ^2^H_WSC_ trend (Abrahim *et al*., 2020). Notably, the relative levels of sucrose and other constituents remained constant in 1N (Fig. 2b).

The inverse relationship between *A*_n_ and δ^2^H_WSC_ in 1N can be more pragmatically explained than in 0N; wherein the higher ratio between photosynthetic rate and respiration led to an accumulation of ^2^H‐depleted sugars not consumed by respiration. That is, there may have been less impact from potential ^2^H‐enrichment of triose phosphates by glyceraldehyde 3‐phosphate (GADPH) in the cytosol, by a reduced relative flux of triose phosphates for respiration (Wieloch *et al*., 2024). This supports the hypothesis that the partitioning of triose phosphates has an associated isotope fractionation influential enough to affect triose phosphate δ^2^H, which in turn affects sugar δ^2^H (Holloway‐Phillips *et al*., 2022). Nonetheless, while GADPH imprints a δ^13^C signal to glucose (Wieloch *et al*., 2021), its role in δ^2^H in sugars still needs to be examined, alongside other potential pathways. Alternatively, if the increase in *A*_n_ was related to a decrease in dark respiration, a decrease in futile sucrose cycling could have reduced H‐exchange with water (Geigenberger & Stitt, 1991; Holloway‐Phillips *et al*., 2022; Lehmann *et al*., 2024b).

Overall, if both needle ages contribute to the phloem pool for cellulose development during their respective measurement periods, the mixed relationships between needle δ^2^H_WSC_ and *A*_n_ complicate the interpretation of tree‐ring δ^2^H, which is known to record tree stress responses (Lehmann *et al*., 2021; Vitali *et al*., 2022, 2023). Therefore, future studies need to trace needle sugar δ^2^H to the phloem to discern the fate of different δ^2^H_WSC_ signals that can vary with needle age and seasonality (Gessler *et al*., 2013).

### Starch δ^2^H has unique relationships to physiological and environmental factors

Starch concentrations and their δ^13^C values show that the dynamic needle starch pool is rapidly used for metabolic processes and is sensitive to resource availability (Yan *et al*., 2012; Desalme *et al*., 2017). These findings are reciprocated by root starch δ^13^C values from the same forest as this study (Tang *et al*., 2022). Based on the dynamic starch concentrations (Fig. 2a) and δ^2^H_starch_ (Fig. 3b) observed here, most of the starch likely originates from newly assimilated carbon. In 0N, most starch was likely transitory because time‐integrated analyses (Fig. 4) revealed that the strongest environmental signals in δ^2^H_starch_ were observed after only 1 (RH, ρ = −0.86) or 2 (δ^2^H_source_, ρ = −0.86) days. Whereas in 1N, starch was likely less dominated by transitory starch, and included a representative portion of long‐term starch storage, as indicated by weaker signals, which were highest after longer integration times than in 0N (e.g. *g*_s_; highest after 5 integration days; ρ = −0.61). These findings are supported by the relatively higher starch concentrations in 1N than in 0N (Fig. 2a). It has been hypothesized that the seasonal δ^2^H_l‐water_ signal in *n*‐alkanes could be obscured by changes in carbohydrate sourcing (Newberry *et al*., 2015). We observed that if a signal in leaf δ^2^H_starch_ is preserved during its remobilization, it does not necessarily dilute the represented time period. Moreover, there was no period of covariability between δ^2^H_alkane_ and δ^2^H_starch_ here (Fig. 3b), implying that needle starch reserves were unlikely to be the main reason for the weak hydrological signals in 0N δ^2^H_alkane_.

This is the first study to identify time‐integrated climatic or physiological signals in δ^2^H_starch_, which were notably different from those of δ^2^H_WSC_ (Fig. 4). For example, 0N δ^2^H_starch_ had a relatively strong signal from RH on the day of sampling, like for 0N δ^2^H_WSC_, but the strongest signal in δ^2^H_WSC_ was *T*:*A*_n_, which was not as prominent in δ^2^H_starch_. Contrary to previous findings, here δ^2^H_starch_ is not necessarily lower than δ^2^H in sugars, in leaves of a C_3_ species (Fig. 2a; Schleucher *et al*., 1999; Schuler *et al*., 2022; Lehmann *et al*., 2024b). For example, in 1N δ^2^H_starch_ was *c*. 18‰ higher than δ^2^H_WSC_ on the same sampling dates. This is likely attributed to seasonal variations *in situ*, since we are the first to measure seasonal δ^2^H_starch_ in a natural forest. When δ^2^H_starch_ was lower than δ^2^H_WSC_, it can be explained by the ^2^H depletion of starch during its synthesis, by the reaction catalyzed by chloroplast phosphoglucose isomerase (EC 5.3.1.9; Schleucher *et al*., 1999). Lehmann *et al*. (2024b) observed that root δ^2^H_starch_ was higher than δ^2^H_WSC_, and this could have been from H isotope exchange between water and intermediates of starch, or by additional sources of isotope fractionation during the metabolic pathway toward starch synthesis in roots. We could extend this to δ^2^H_starch_ here, if there were substantial enough intermediaries during its synthesis. Following on, the steep decrease in δ^2^H_starch_ at the start of 0N sampling could result from a shift from heterotrophy to autotrophy, as observed in new needle growth of *Pinus pisaster* by Desalme *et al*. (2017). On the sampling dates following the steep δ^2^H_starch_ decrease, δ^2^H_starch_ did not have a noticeably different trend from δ^2^H_WSC_ (Fig. 2b). Nonetheless, the overall differences in δ^2^H_starch_ and δ^2^H_WSC_ were enough to lead to unique physiological and environmental signals in δ^2^H_starch_, which were different from those for δ^2^H_WSC_. The unique signals in δ^2^H_starch_ could obscure the signal in δ^2^H_WSC_, in addition to isotope fractionation by starch remobilization.

### The necessity of time scales for interpreting δ^2^H bioindicators

It has been previously shown that δ^2^H_l‐water_ heterogeneity is related to δ^2^H_alkane_ heterogeneity within a leaf (Zhu *et al*., 2020; Liu *et al*., 2021) and that photosynthetic water is likely more exposed to evaporative enrichment (Baca Cabrera *et al*., 2023). In our study, such heterogeneity also includes the effects of leaf water compartmentation between the chloroplast and cytosol (Baca Cabrera *et al*., 2023). Our results suggest that needle water heterogeneity does not substantially affect the relationship between δ^2^H_alkane_ and bulk δ^2^H_n‐water_ over a timescale of multiple weeks (Fig. 6). This could be due to increased δ^2^H_source_ variability over multiple weeks counteracting the role of leaf water heterogeneity (Fig. 3a). For 1N δ^2^H_WSC_, leaf water heterogeneity became more relevant because δ^2^H_WSC_ was more strongly related to ∆^2^H_n‐water_ than to δ^2^H_n‐water_ (Fig. 4). This could be because the shorter time integration for δ^2^H_WSC_ did not allow δ^2^H_source_ to vary enough to be more influential than within‐needle isotope heterogeneity. Therefore, while our results are based on moderately performing model gap filling for δ^2^H_n‐water_ and ∆^2^H_n‐water_ (Figs 3a, S5; Table S1), they build on existing evidence (Zhu *et al*., 2020; Baca Cabrera *et al*., 2023), suggesting that it is worthwhile to further explore δ^2^H_n‐water_ heterogeneity effects on the prevailing information stored in δ^2^H of leaf carbohydrates and thus tree rings. Overall, its different roles to δ^2^H_alkane_ and δ^2^H_WSC_ suggest that the integration time has the potential to affect not only the period that δ^2^H represents but also the factors most influential to δ^2^H.

### Conclusion

By investigating δ^2^H_alkane_, δ^2^H_WSC_ and δ^2^H_starch_ in a natural forest, this study has bridged the understanding between leaf‐level biosynthetic ^2^H‐fractionation, and paleoclimatic reconstructions. We have demonstrated that it is critical to use time‐integrated analyses when exploring physiological and environmental signals in organic compound δ^2^H at the leaf level, especially for *n*‐alkanes. Our results indicate that the prevailing physiological signal in leaf sugars could be a mixed signal from different needle ages or seasonal stages, with potential interference from remobilization of δ^2^H_source_‐ or *g*_s_‐signals in δ^2^H_starch_. Thus, it is important to investigate the prevailing information transported by δ^2^H in sugars to the phloem. Finally, we demonstrate that *de novo* mature needle δ^2^H_alkane_ synthesis in the field is substantial enough to establish a weak relationship with processes correlated with hydrological factors (e.g. RH, δ^2^H_source_ and δ^2^H_n‐water_), making it potentially more reliable than 0N δ^2^H_alkane_, which is exposed to more variable biosynthetic isotope fractionation. This finding is promising for paleoclimatic reconstructions, because δ^2^H_alkane_ delivered in fallen leaves could possess an integrated palaeohydrological signal from the leaf lifespan. However, we still lack information, to which extent fallen and living leaves contribute to the soil δ^2^H_alkane_ reservoir, a topic that should be targeted in future studies.

## Competing interests

None declared.

## Author contributions

CA, KTR‐G, GLBW, MML and MS were involved in conceptualization. YT, PPS‐A, KTR‐G and ES were involved in fieldwork. CA, GLBW, TCS, MML, MS and YT were involved in laboratory work. CA, GLBW, KTR‐G, ES, MML, MS and YT were involved in data quality control. CA was involved in visualization, and modeling and statistical analysis. CA, YT and TCS were involved in writing – original draft. KTR‐G, GLBW, MML, YT, TCS, MS, ES, PPS‐A and CA were involved in writing – review and editing. KTR‐G was involved in supervision. CA, KTR‐G, GLBW, MML and MS were involved in funding acquisition.

## Disclaimer

The New Phytologist Foundation remains neutral with regard to jurisdictional claims in maps and in any institutional affiliations.

## Supporting information


**Fig. S1** Transpiration rate measured from two gas cuvette exchange systems with gap filling by model replication.
**Fig. S2** Successfully replicated source δ^18^O predictions from Leppä *et al*. (2022).
**Fig. S3** Relationships between measured δ^2^H and δ^18^O in source water and water vapor.
**Fig. S4** Relationships between oxygen‐18 (δ^18^O, ‰) and deuterium (δ^2^H, ‰) for modeled and measured soil water and twig water.
**Fig. S5** Quality of different isotope modeling for leaf water heavy isotope values and enrichment when measured δ^2^H data for source water and water vapor are missing.
**Methods S1** Description for extraction of water‐soluble carbohydrates and starch.
**Methods S2** Description of isotope modeling to implement in time‐integrated analyses.
**Table S1** Regression fits showing the performance of different isotope modeling for leaf water heavy isotope values and enrichment when measured δ^2^H data for source water and water vapor are missing.
**Table S2** Mean biosynthetic hydrogen isotope fractionation between modeled leaf water and measured leaf organic compounds.
Please note: Wiley is not responsible for the content or functionality of any Supporting Information supplied by the authors. Any queries (other than missing material) should be directed to the *New Phytologist* Central Office.

## Data Availability

The novel data that support the findings of this study are available on Dryad doi: 10.5061/dryad.xpnvx0krq. The open‐access data from SMEAR and ICOS are accessible via their data portals; ‘https://smear.avaa.csc.fi/download' and ‘https://data.icos‐cp.eu/portal/', respectively.
