# Supplementary material for: Time‐integrated δ2 H in n‐alkanes and carbohydrates from boreal needles reveal intra‐annual physiological and environmental signals
Source: New Phytol. 2025 Feb 21;246(2):498–514. doi: 10.1111/nph.20448 (PMC11923416; doi:10.1111/nph.20448)
Supplement: Supplementary file 1 — Fig. S1 Transpiration rate measured from two gas cuvette exchange systems with gap filling by model replication. Fig. S2 Successfully replicated source δ18O predictions from Leppä et al. (2022). Fig. S3 Relationships between measured δ2H and δ18O in source water and water vapor. Fig. S4 Relationships between oxygen‐18 (δ18O, ‰) and deuterium (δ2H, ‰) for modeled and measured soil water and twig water. Fig. S5 Quality of different isotope modeling for leaf water heavy isotope values and enrichment when measured δ2H data for source water and water vapor are missing. Methods S1 Description for extraction of water‐soluble carbohydrates and starch. Methods S2 Description of isotope modeling to implement in time‐integrated analyses. Table S1 Regression fits showing the performance of different isotope modeling for leaf water heavy isotope values and enrichment when measured δ2H data for source water and water vapor are missing. Table S2 Mean biosynthetic hydrogen isotope fractionation between modeled leaf water and measured leaf organic compounds. Please note: Wiley is not responsible for the content or functionality of any Supporting Information supplied by the authors. Any queries (other than missing material) should be directed to the New Phytologist Central Office. [file NPH-246-498-s001.pdf]

***New Phytologist* Supporting Information**

Article title: **Time-integrated δ^2H in *n*-alkanes and carbohydrates from boreal needles reveal intra-annual physiological and environmental signals**

Authors: Charlotte Angove, Guido L.B. Wiesenberg, Marco M. Lehmann, Matthias Saurer, Yu Tang, Elina Sahlstedt, Tatjana C. Speckert, Pauliina P. Schiestl-Aalto & Katja T. Rinne-Garmston

Article acceptance date: 19 December 2024

The following Supporting Information is available for this article:

Fig. S1 Transpiration rate measured from two gas cuvette exchange systems with gap filling by model replication.

Methods S1 Description for extraction of water-soluble carbohydrates and starch.

Methods S2 Description of isotope modelling to implement in time-integrated analyses.

Fig. S2 Successfully replicated source δ^{18}O predictions from Leppä *et al.* (2022).

Fig. S3 Relationships between measured δ^2H and δ^{18}O in source water and water vapor.

Fig. S4 Relationships between oxygen-18 (δ^{18}O, ‰) and deuterium (δ^2H, ‰) for modelled and measured soil water and twig water.

Table S1 Regression fits showing the performance of different isotope modelling for leaf water heavy isotope values and enrichment when measured δ^2H data for source water and water vapor are missing.

Fig. S5 Quality of different isotope modelling for leaf water heavy isotope values and enrichment when measured δ^2H data for source water and water vapor are missing.

Table S2 Mean biosynthetic hydrogen isotope fractionation between modelled leaf water and measured leaf organic compounds.

Fig. S1 Successfully replicated transpiration rate (E, mol m^{-2} s^{-1}) predictions from Leppä et al. (2022), for five *Pinus sylvestris* trees in Hyytiälä Forest, central Finland, during the 2018-2019 growing seasons. Scatter plot represents daily means when measured data is available, while the line graph shows data averaged 9-15h. Seasonal variability largely superseded differences in needle age; chamber 1 measured one year-old needle gas exchange, while chamber 2 measured two year-old needle gas exchange.

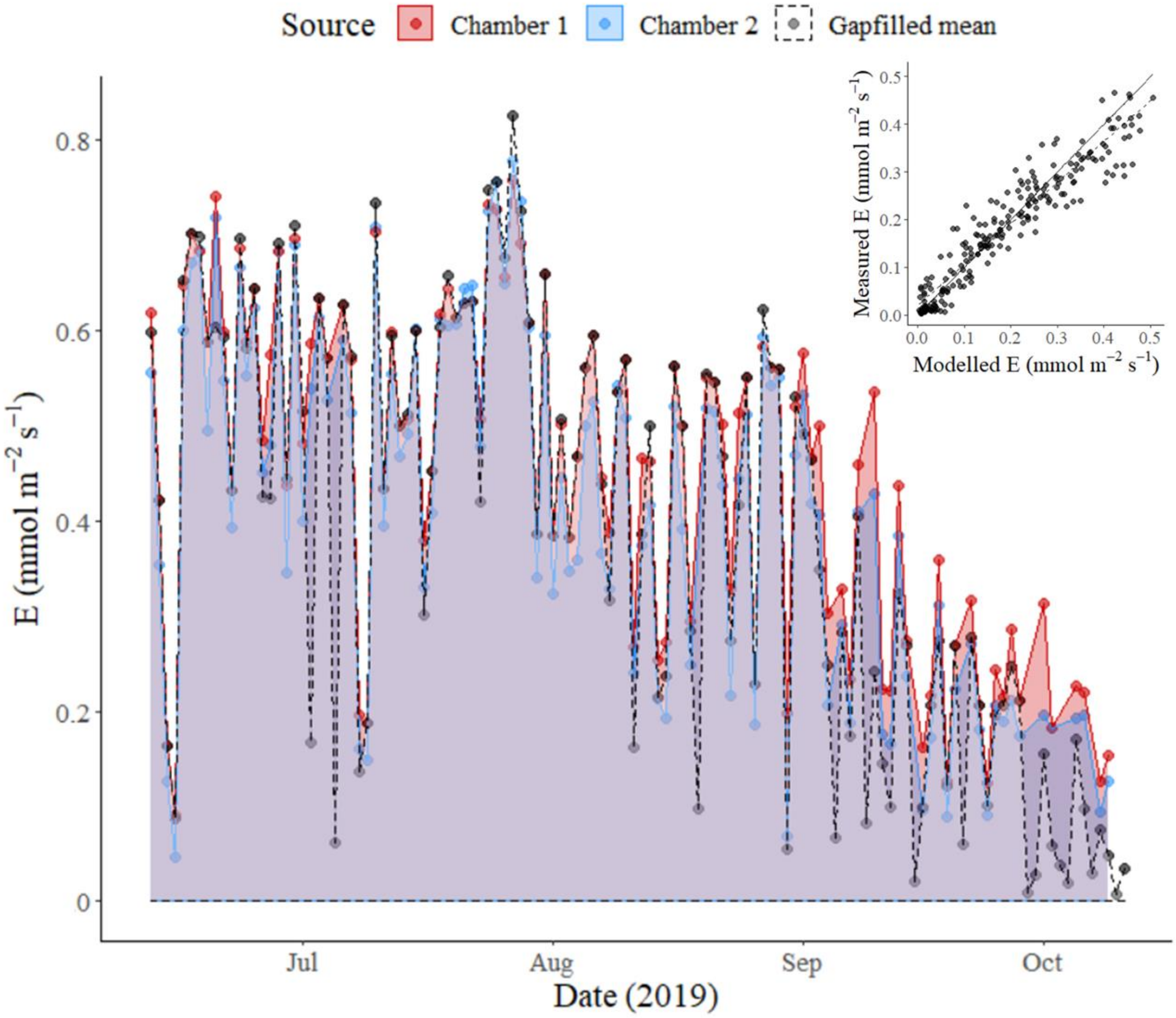

Methods S1 Description for extraction of water-soluble carbohydrates and starch.

Extraction of water-soluble carbohydrates

WSC were extracted from needle powder using the hot water extraction method at 85°C for 30 min, following the procedure outlined by Wanek *et al.* (2001). The separated supernatant was subsequently purified using three types of sample treatment cartridges, which removed amino acids, organic acids and phenolic compounds (Rinne *et al.*, 2012). The purified samples were freeze-dried, dissolved in deionized water, filtered through a 0.45 µm syringe filter (Acrodisc; Pall Corp., Port Washington, NY, USA) and stored at −20°C. It was possible to measure a portion of samples for compound-specific concentrations of sugars and sugar alcohols (i.e., pinitol), using an irMS (Delta V Advantage, Thermo Fisher Scientific) coupled to a high-performance liquid chromatography (HPLC) set-up with an LC-IsoLINK interface (Finnigan), then their concentrations were inferred by comparison between peak areas and the associated carbon content of compound-specific standards (Rinne *et al.*, 2012). We subsequently calculated the bulk WSC concentrations as the sum of compound-specific concentrations, and gap filled sampling dates without compound-specific measurements using the weight of the sample before and after hot water extraction.

Extraction of starch

To eliminate lipids, the pellet was washed four times with 1.2 ml methanol-chloroform-water solution (12:5:3, v/v/v) and with 1.2 ml deionized water in between. The lipid-free pellet was re-suspended in 0.75 ml deionized water, boiled at 99 °C for 15 minutes to gelatinize the starch, and then hydrolyzed at 85 °C for 2 hours with the addition of 0.25 ml purified (by Vivaspin 15R, Sartorius, Göttingen, Germany) α-amylase solution (3000 U ml^{-1}, Sigma-Aldrich, Buchs, Switzerland). The hydrolyzed starch was separated from enzymatic residues using centrifugation filters (Vivaspin 500, Sartorius) and stored at –20 °C. When starch concentrations were low, samples from multiple trees were pooled then concentrated (desiccated in freeze-dryer then dissolved in a fixed volume of solute) to achieve a δ^2H measurement.

Methods S2 Description for extraction of water-soluble carbohydrates and starch.

To gapfill δ^2H_{vapor} and δ^2H_{source} measurements at daily resolution, we trialed two different approaches. Both are based on a mass-balance based model for the rooting zone water budget:

$$\frac{dR_s W_{soil}}{dt} = R_{rain}P - R_s E_{tot} - R_s D \qquad \text{(Equation S1)}$$

(Ogée *et al.*, 2009), where R_s is the isotopic ratio of source water (oxygen or hydrogen), W_{soil} (kg m^{-2}) is the water content of the rooting zone, R_{rain} is the isotopic ratio of rain, P is precipitation (kg m^{-2} s^{-1}), E_{tot} is total evapotranspiration (kg m^{-2} s^{-1}) and D is drainage (kg m^{-2} s^{-1}). This model has already been applied to successfully predict $\delta^{18}O$ in source water ($\delta^{18}O_{source}$) at the study site during 2018 and 2019 by Leppä *et al.* (2022), and we accurately replicated their predictions by following their demonstration (Fig. **S2**). Our first approach to gapfill δ^2H_{source} measurements, was directly modelling it using Equation S1, by replacing the R_{rain} for oxygen with its equivalent for hydrogen, measured at monthly resolution at the study site. A limitation of using this model is that it assumes there is no isotope fractionation during tree water uptake (Ogée *et al.*, 2009), which brings a substantial risk of error in δ^2H_{source} prediction accuracy because evidence suggests H isotopes can fractionate during water uptake (Lin & Sternberg, 1992; Ellsworth & Williams, 2007; Zhao *et al.*, 2016, 2024; Newberry *et al.*, 2017; Vargas *et al.*, 2017; Barbeta *et al.*, 2019, 2020). Therefore, we tested a second modelling approach suggested by Zhao *et al.* (2024), where we inferred δ^2H_{source} from predicted $\delta^{18}O_{source}$ (Equation S1) using their close, measured relationship (R^2(M) = 0.87, $p < 0.01$, Fig. **S3a**). Overall, the first δ^2H_{source} modelling approach assumed that water uptake was not isotope fractionating (hereafter 'modelled soil water δ^2H'), while the second approach assumed it was hydrogen isotope fractionating (hereafter 'modelled twig water δ^2H'). The best-performing approach was the latter ($\delta^2H_{source} = \delta^{18}O_{source} \times 6.53 - 20.55$; Fig. **S4a**). It was unlikely that model outcomes were substantially affected by source water sampling bias, such as isotope fractionation during cryogenic water extraction (Chen *et al.* 2020), because the model assuming hydrogen isotope fractionation during tree water uptake was not only the best predictor for δ^2H_{source}, but also for needle water δ^2H (Fig. **S4b**), which is not prone to the same sampling bias issues as δ^2H_{source}.

δ^2H_{vapor} was inferred using its $\delta^{18}O$ equivalent replicated from Leppä *et al.* (2022), using the close measured relationship between δ^2H and $\delta^{18}O$ in vapor (R^2= 0.94, $p < 0.01$, Fig. **S3b**). The vapor

$\delta^{18}O$ data was a six-hourly prediction sourced from IsoGSM, an isotope-enabled atmospheric circulation model (Yoshimura *et al.*, 2008, 2011).

Finally, we modelled $\delta^2H_{n\text{-water}}$ and $\Delta^2H_{n\text{-water}}$ using the following model variations of the Craig Gordon model: The baseline Craig Gordon model as described and demonstrated by Cernusak *et al.* (2016); the model with a two-pool correction (Two-pool value (ϕ): 0.316 (Roden *et al.*, 2015)) or Péclet correction (Effective path length (*L*): Calculated using relationship between transpiration rate and *L* (Song *et al.*, 2013)) as described by Angove *et al.* (2023); and the model with a non-steady state correction as described and demonstrated by Leppä *et al.* (2022), except that fractionation factors for ^{18}O were replaced with those for ^2H (Cernusak *et al.* 2016). Predictions were at half-hourly resolution, based on half-hourly meteorological and leaf gas exchange data, with 6-hourly δ^2H_{vapor} and daily δ^2H_{source} estimates. $\delta^2H_{n\text{-water}}$ and $\Delta^2H_{n\text{-water}}$ predictions were summarized for daily averages (9h-15h) and compared to measured $\delta^2H_{n\text{-water}}$ and $\Delta^2H_{n\text{-water}}$, respectively (Fig. **S5**, Table **S1**). The time window used for daily average model evaluation did not noticeably influence model diagnostics, owing to the strong role of low-resolution δ^2H_{vapor} and δ^2H_{source} (6-hourly/daily). We used measured δ^2H_{vapor} and δ^2H_{source} when available, and their modelled values gap filled missing data. We selected the Craig Gordon model with a Péclet correction for this study because it accurately predicted $\Delta^2H_{n\text{-water}}$ when measured δ^2H_{source} and δ^2H_{vapor} were available (Angove *et al.*, 2023), and it was the only model variation to moderately predict $\Delta^2H_{n\text{-water}}$ when measured δ^2H_{source} and δ^2H_{vapor} were not available (Table **S1**, Fig. **S5**).

Fig. S2 Successfully replicated source water $\delta^{18}O$ ($\delta^{18}O_{source}$) predictions from Leppä et al. (2022), for five *Pinus sylvestris* trees in Hyytiälä Forest, central Finland, during the 2018-2019 growing seasons. Solid circles represent twig water $\delta^{18}O$ while open triangles represent soil water $\delta^{18}O$. In (a) and (b), the solid line represents modelled $\delta^{18}O_{source}$, while in (c) the solid line shows a 1:1 ratio and the dashed line shows the significant linear mixed model fit. R^2(M) is the linear mixed model's marginal R^2, RMSE is Root Mean Square Error, and ICC is Intraclass Correlation.

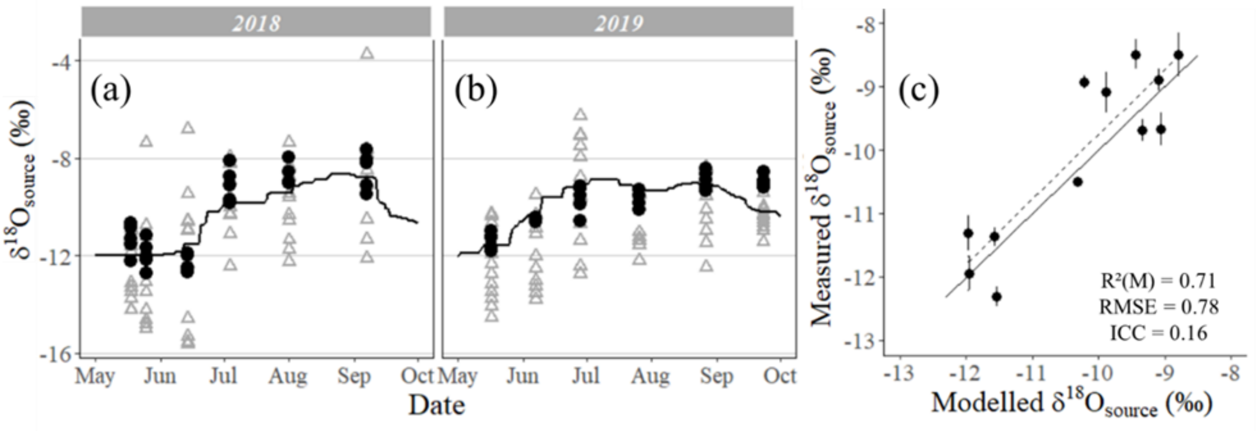

Fig. S3 Relationships between measured δ^2H and $\delta^{18}O$, (a) in source water, and (b) in water vapor. In (a), datapoints represent means of five *Pinus sylvestris* trees and error bars show standard error. The solid lines denote the meteoric water line, while the dashed lines show significant relationships (a: $\delta^2H = \delta^{18}O \times 6.53 - 20.55$, Marginal $R^2 = 0.87$, ICC = 0.06; b: $\delta^2H = \delta^{18}O \times 7.23 + 21.84$; $R^2 = 0.94$).

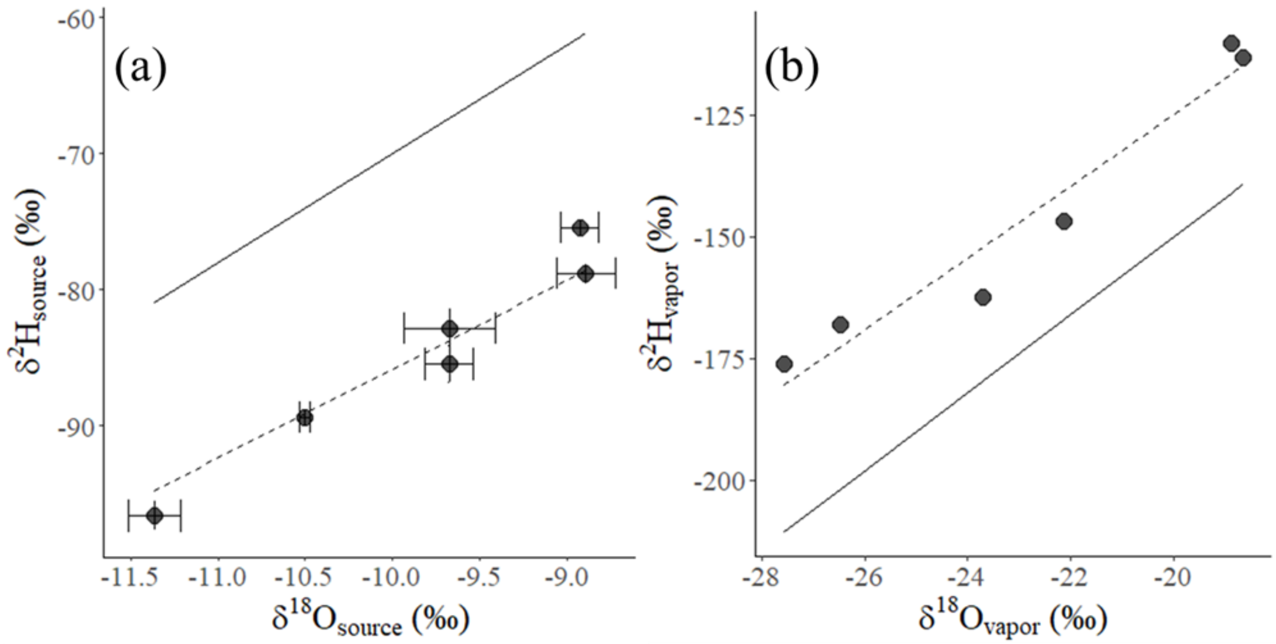

Fig. S4 Performance of different approaches to model source water δ^2H (δ^2H_{source}) in *Pinus sylvestris*, using a mass-balanced model for water in the rooting zone. Twig-water based modelling assumes fractionation of hydrogen isotopes during tree water uptake, while soil-water based modelling does not. (a) Relationships between $\delta^{18}O$ and δ^2H (‰). The solid line shows the local meteoric water line (LMWL) calculated from measured precipitation δ^2H and $\delta^{18}O$ at the time of sampling. The dashed line shows the linear mixed model fit between measured twig water δ^2H and $\delta^{18}O$. (b) Needle water δ^2H ($\delta^2H_{n\text{-}water}$) model accuracy from the twig-water and soil-water based δ^2H_{source} predictions, with Root Mean Square Error (RMSE) diagnostics (CG is the Craig Gordon model; CG × P is CG with a Péclet correction; CG(1- ϕ) is CG with a two-pool correction).

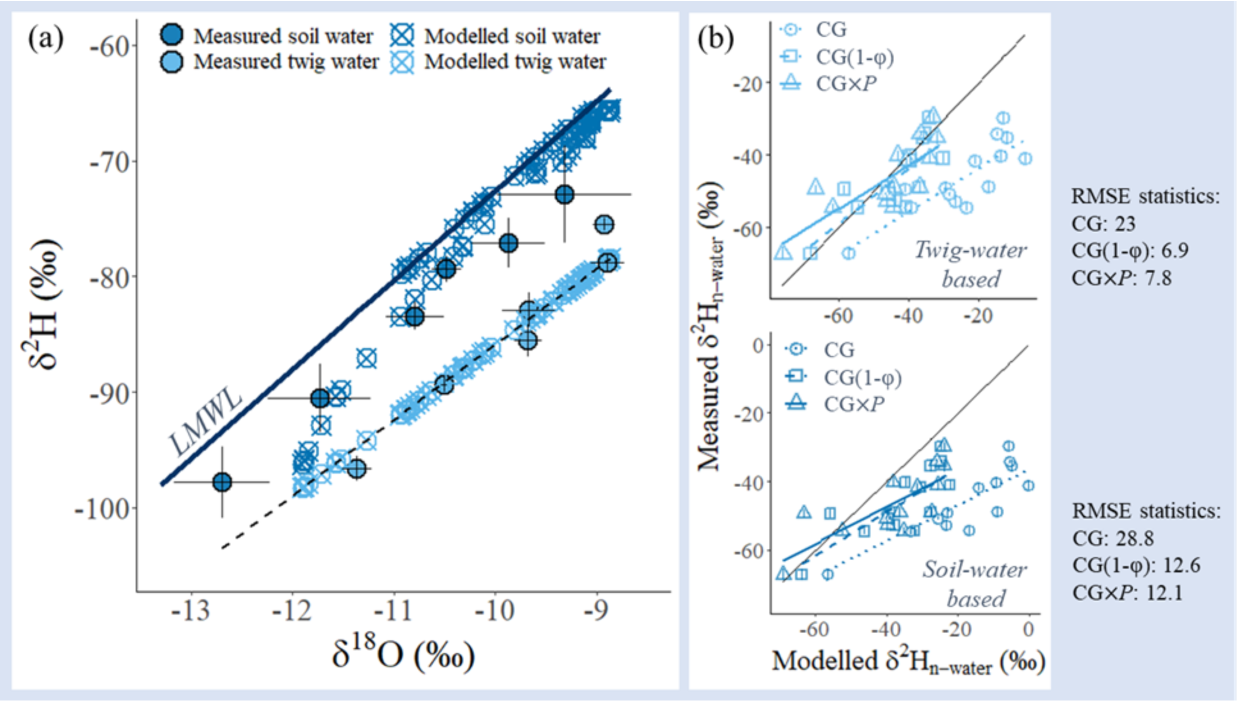

Table S1 Regression fits that evaluate the performance of different approaches for modelling daily-averaged needle water isotope variability for *Pinus sylvestris*: δ^2H ($\delta^2H_{n\text{-water}}$), $\delta^{18}O$ ($\delta^{18}O_{n\text{-water}}$), ^2H enrichment ($\Delta^2H_{n\text{-water}}$) and ^{18}O enrichment ($\Delta^{18}O_{n\text{-water}}$); using different corrections on the Craig Gordon (CG) model: where NSS is CG with a non-steady state correction, CG × P is CG with a Péclet correction, $CG(1-\varphi)$ is CG with a two-pool correction.

	Intercept	Slope[a]	*F*	R^2	RMSE
$\delta^2H_{n\text{-water}}$ predictions related to observed $\delta^2H_{n\text{-water}}$					
CG	-32.84 ± 3.9	**0.58** ± 0.15	15.54	0.56	24.55
NSS	-32.81 ± 3.89	**0.58** ± 0.15	15.64	0.57	24.52
CG × P	-18.77 ± 7.08	**0.61** ± 0.15	16.28	0.58	7.6
$CG(1-\varphi)$	-15.58 ± 8.91	**0.72** ± 0.2	12.57	0.51	8.31
$\delta^{18}O_{n\text{-water}}$ predictions related to observed $\delta^{18}O_{n\text{-water}}$					
CG	-0.85 ± 0.7	**0.78** ± 0.07	109.8	0.9	3.39
NSS	-0.2 ± 0.58	**0.8** ± 0.07	148	0.93	2.56
CG × P	3.64 ± 0.62	**1.1** ± 0.12	83.27	0.87	4.47
$CG(1-\varphi)$	2.69 ± 0.59	**1.1** ± 0.11	94.45	0.89	3.7
$\Delta^2H_{n\text{-water}}$ predictions related to observed $\Delta^2H_{n\text{-water}}$					
CG	-14.56 ± 48.35	0.85 ± 0.65	1.71	0.3	28.71
NSS	-14.52 ± 48.24	0.85 ± 0.65	1.72	0.3	28.7
CG × P	-46.13 ± 16.57	**1.92** ± 0.33	33.05	0.89	8.22
$CG(1-\varphi)$	-14.56 ± 48.35	1.24 ± 0.95	1.71	0.3	12.27
$\Delta^{18}O_{n\text{-water}}$ predictions related to observed $\Delta^{18}O_{n\text{-water}}$					
CG	-4 ± 3.97	**1.05** ± 0.19	31.25	0.89	3.5
NSS	-1.16 ± 1.93	**0.97** ± 0.1	102.1	0.96	2.21
CG × P	-0.87 ± 2.8	**1.4** ± 0.2	46.84	0.92	4.96
$CG(1-\varphi)$	-4 ± 3.97	**1.53** ± 0.27	31.25	0.89	4.31

[a]*Bold when significant ($p < 0.05$)*

Fig. S5 Quality of different modelling approaches for leaf water isotope variability. (a) Leaf water ^{2}H enrichment above source water ($\Delta^2H_{n\text{-}water}$), (b) ^{18}O enrichment above source water ($\Delta^{18}O_{n\text{-}water}$), (c) bulk leaf water δ^2H ($\delta^2H_{n\text{-}water}$), and bulk leaf water δ^{18}O ($\delta^{18}O_{n\text{-}water}$). Measured values are means (± standard error) of 3 – 5 *Pinus sylvestris* trees measured during 2019 at Hyytiälä Forest, central Finland. Dashed lines show regression fits when significant, while coloured symbols show results from different leaf water isotope model corrections (CG = Craig Gordon model, $CG(1-\varphi)$ = CG with two-pool correction, NSS = CG with non-steady state correction, $CG \times P$ = CG with Péclet correction).

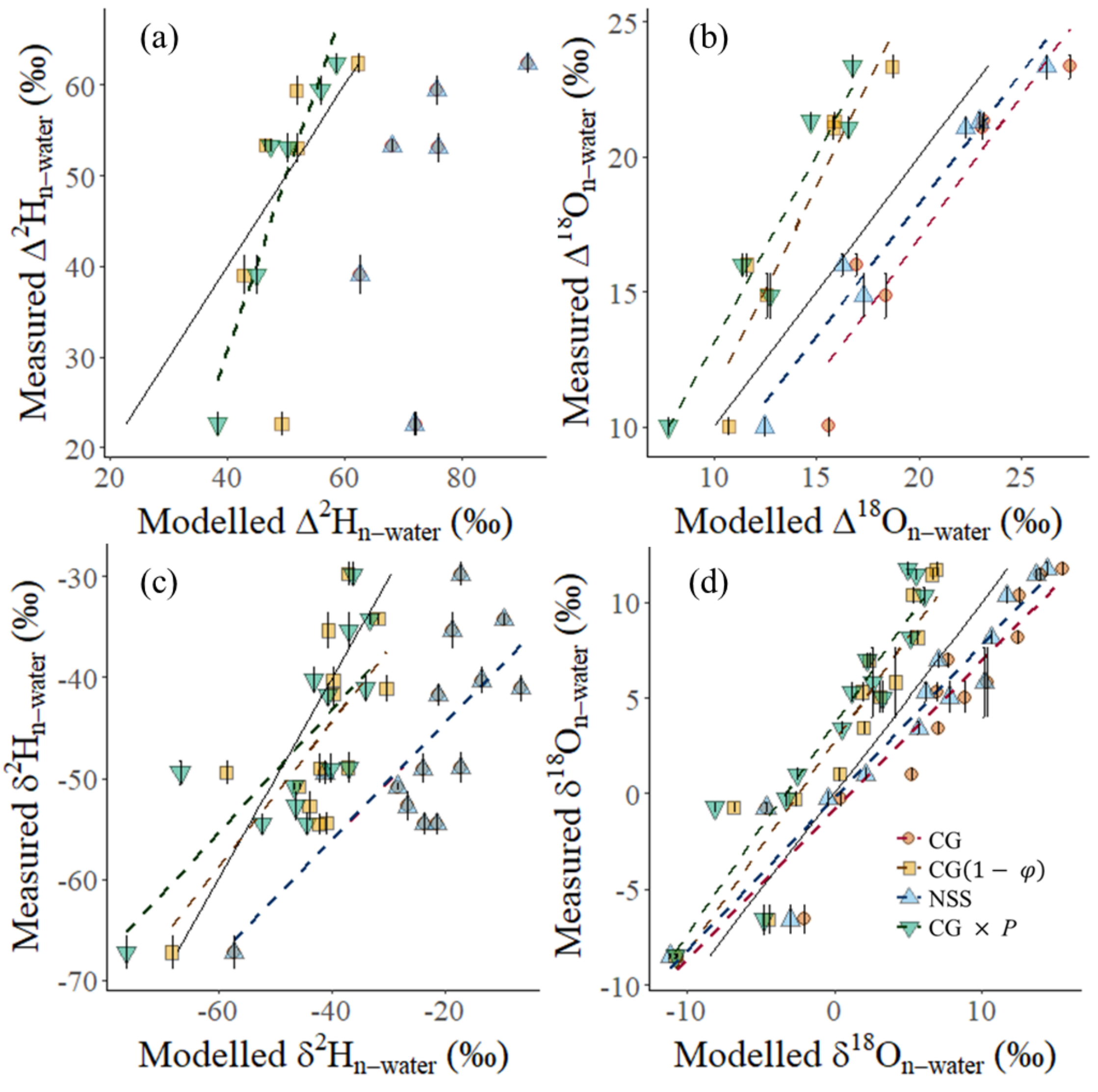

Table S2 Mean biosynthetic hydrogen isotope fractionation (Equation 4 in Holloway-Phillips et al. 2022) from modelled leaf water (Craig Gordon model with Péclet correction) to measured leaf organic compounds in 5 *Pinus sylvestris* trees at Hyytiälä Forest, central Finland.

	Current-year needles	One year-old needles
Water-soluble carbohydrates	2.89	3.1
Starch	3.41	2.89
n-Alkanes	3.07	3.41

References

Angove C, Lehmann MM, Saurer M, Tang Y, Kilpeläinen P, Kahmen A, Schiestl-Aalto PP, Tikkasalo OP, Bäck JK, Rinne-Garmston KT. 2023. Unsaturated intercellular vapor pressure is relevant for leaf water heavy isotope enrichment. *bioRxiv*. doi: 10.1101/2023.09.12.557463.

Barbeta A, Sam PJ, Clavé L, Wingate L, Gimeno TE, Frejaville B, Wohl S, Ogee J. 2019. Unexplained hydrogen isotope offsets complicate the identification and quantification of tree water sources in a riparian forest. *Hydrology and Earth System Sciences*, 23: 1–31.

Cernusak LA, Barbour MM, Arndt SK, Cheesman AW, English NB, Feild TS, Helliker BR, Holloway-Phillips MM, Holtum JA, Kahmen A et al. 2016. Stable isotopes in leaf water of terrestrial plants. *Plant Cell and Environment*. doi: 10.1111/pce.12703.

Chen Y, Helliker BR, Tang X, Li F, Zhou Y, Song X. 2020. Stem water cryogenic extraction biases estimation in deuterium isotope composition of plant source water. *Proceedings of the National Academy of Sciences of the United States of America*. 117: 33345-50.

Ellsworth PZ, Williams DG. 2007. Hydrogen isotope fractionation during water uptake by woody xerophytes. *Plant and Soil* 291: 93–107.

Holloway-Phillips M, Baan J, Nelson DB, Lehmann MM, Tcherkez G, Kahmen A. 2022. Species variation in the hydrogen isotope composition of leaf cellulose is mostly driven by isotopic variation in leaf sucrose. *Plant Cell and Environment*. doi: 10.1111/pce.14362.

Leppä K, Tang Y, Ogée J, Launiainen S, Kahmen A, Kolari P, Sahlstedt E, Saurer M, Schiestl-Aalto P, Rinne-Garmston KT. 2022. Explicitly accounting for needle sugar pool size crucial for predicting intra-seasonal dynamics of needle carbohydrates $\delta^{18}O$ and $\delta^{13}C$. *New Phytologist*. doi: 10.1111/nph.18227.

Lin GH, Sternberg LSL. 1992. Comparative study of water uptake and photosynthetic gas exchange between scrub and fringe red mangroves, Rhizophora mangle L. *Oecologia*. 90: 399–403.

Newberry SL, Nelson DB, Kahmen A. 2017. Cryogenic vacuum artifacts do not affect plant water-uptake studies using stable isotope analysis. *Ecohydrology.* 10: e1892.

Ogée J, Barbour MM, Wingate L, Bert D, Bosc A, Stievenard M, Lambrot C, Pierre M, Bariac T, Loustau D, Dewar RC. 2009. A single-substrate model to interpret intra-annual stable isotope signals in tree-ring cellulose. *Plant, Cell & Environment*. 32: 1071–1090.

Rinne KT, Saurer M, Streit K, Siegwolf RT. 2012. Evaluation of a liquid chromatography method for compound-specific $\delta^{13}C$ analysis of plant carbohydrates in alkaline media. *Rapid Communications in Mass Spectrometry*. doi: 10.1002/rcm.6334.

Roden J, Kahmen A, Buchmann N, Siegwolf R. 2015. The enigma of effective path length for 18O enrichment in leaf water of conifers. *Plant Cell and Environment*. doi: 10.1111/pce.12568.

Song X, Barbour MM, Farquhar GD, Vann DR, Helliker BR. 2013. Transpiration rate relates to within- and across-species variations in effective path length in a leaf water model of oxygen isotope enrichment. *Plant, Cell and Environment*. doi: 10.1111/pce.12063.

Vargas AI, Schaffer B, Yuhong L, Sternberg LDL. 2017. Testing plant use of mobile and immobile soil water sources using stable isotope experiment. *New Phytologist.* 215: 582–594.

Wanek W, Heintel S, Richter A. 2001. Preparation of starch and other carbon fractions from higher plant leaves for stable carbon isotope analysis. *Rapid Communications in Mass Spectrometry*. doi: 10.1002/rcm.353.

Yoshimura K, Kanamitsu M, Noone D, Oki T. 2008. Historical isotope simulation using Reanalysis atmospheric data. *Journal of Geophysical Research Atmospheres*. doi: 10.1029/2008JD010074.

Yoshimura K, Frankenberg C, Lee J, Kanamitsu M, Worden J, Röckmann T. 2011. Comparison of an isotopic atmospheric general circulation model with new quasi-global satellite measurements of water vapor isotopologues. *Journal of Geophysical Research Atmospheres*. doi: 10.1029/2011JD016035.

Zhao LJ, Wang LX, Cernusak LA, Liu XH, Xiao HL, Zhou MX, Zhang SQ. 2016. Significant difference in hydrogen isotope composition between xylem and tissue water in Populus euphratica. *Plant, Cell & Environment.* 39: 1848–1857.

Zhao L, Liu X, Wang N, Barbeta A, Zhang Y, Cernusak LA, Wang L. 2024. The determining factors of hydrogen isotope offsets between plants and their source waters. *New Phytologist.* 241:2009-2024.
